# An optimized Western blot assay provides a comprehensive assessment of the physiological endoproteolytic processing of the prion protein

**DOI:** 10.1016/j.jbc.2022.102823

**Published:** 2022-12-22

**Authors:** Ilaria Vanni, Floriana Iacobone, Claudia D’Agostino, Matteo Giovannelli, Laura Pirisinu, Hermann Clemens Altmeppen, Joaquin Castilla, Juan Maria Torres, Umberto Agrimi, Romolo Nonno

**Affiliations:** 1 Department of Food Safety, Nutrition and Veterinary Public Health, Istituto Superiore di Sanità, Rome, Italy; 2 Institute of Neuropathology, University Medical Center Hamburg-Eppendorf (UKE), Hamburg, Germany; 3 Basque Research and Technology Alliance (BRTA) - CIC BioGUNE & IKERBasque, Bizkaia, Spain; 4 Centro de Investigación Biomédica en Red de Enfermedades infecciosas (CIBERINFEC), Instituto de Salud Carlos III, Madrid, Spain; 5 Centro de Investigación en Sanidad Animal (CISA-INIA-CSIC), Valdeolmos, Madrid, Spain

**Keywords:** ADAM, prion, prion disease, neurodegenerative disease, proteolysis, shedding, protein misfolding, PrPC, proteoform, Western blot, ADAM, A Disintegrin And Metalloproteinase, C1, C-terminal GPI-anchored cellular prion proteoform derived by α-cleavage, C2, C-terminal GPI-anchored cellular prion proteoform derived by β-cleavage, C3 and C3′, C-terminal GPI-anchored cellular prion proteoforms derived by γ-cleavage, FL, full length, FL-PrP^C^, full-length GPI-anchored cellular prion proteoform, GPI, glycophosphatidylinositol, mAb, monoclonal antibody, MW, molecular weight, N1, released N-terminal cellular prion proteoform derived by α-cleavage, N2, released N-terminal cellular prion proteoform derived by β-cleavage, N3 and N3′, released N-terminal cellular prion proteoforms derived by γ-cleavage, PrP^C^, cellular prion protein, sC1, shed C1, sFL, shed FL-PrP^C^, TSE, transmissible spongiform encephalopathy, WB, Western blotting

## Abstract

The prion protein (PrP^C^) is subjected to several conserved endoproteolytic events producing bioactive fragments that are of increasing interest for their physiological functions and their implication in the pathogenesis of prion diseases and other neurodegenerative diseases. However, systematic and comprehensive investigations on the full spectrum of PrP^C^ proteoforms have been hampered by the lack of methods able to identify all PrP^C^-derived proteoforms. Building on previous knowledge of PrP^C^ endoproteolytic processing, we thus developed an optimized Western blot assay able to obtain the maximum information about PrP^C^ constitutive processing and the relative abundance of PrP^C^ proteoforms in a complex biological sample. This approach led to the concurrent identification of the whole spectrum of known endoproteolytic-derived PrP^C^ proteoforms in brain homogenates, including C-terminal, N-terminal and, most importantly, shed PrP^C^-derived fragments. Endoproteolytic processing of PrP^C^ was remarkably similar in the brain of widely used wild type and transgenic rodent models, with α-cleavage-derived C1 representing the most abundant proteoform and ADAM10-mediated shedding being an unexpectedly prominent proteolytic event. Interestingly, the relative amount of shed PrP^C^ was higher in WT mice than in most other models. Our results indicate that constitutive endoproteolytic processing of PrP^C^ is not affected by PrP^C^ overexpression or host factors other than PrP^C^ but can be impacted by PrP^C^ primary structure. Finally, this method represents a crucial step in gaining insight into pathophysiological roles, biomarker suitability, and therapeutic potential of shed PrP^C^ and for a comprehensive appraisal of PrP^C^ proteoforms in therapies, drug screening, or in the progression of neurodegenerative diseases.

The cellular prion protein (PrP^C^) is mainly known for its pivotal role in transmissible spongiform encephalopathies (TSEs), or prion diseases, which are fatal neurodegenerative diseases affecting humans and animals. These unusual diseases are associated with the central nervous system accumulation of autocatalytically self-replicating aggregates of PrP^Sc^, a misfolded pathological isoform of the host-encoded PrP^C^ (1). PrP^C^ is a cell surface glycoprotein of 209 amino acids, encoded by the *Prnp* gene. Despite the translation of a single polypeptide, the protein undergoes several post-translational modifications, that is, the addition of N-linked sugars at residues 181 and 197, a glycophosphatidylinositol (GPI) anchor for membrane attachment and a disulphide bond (2, 3), as well as constitutive processing at various cellular locations, which widen the number of its physiological active forms. We will thus refer to all these forms as PrP^C^ proteoforms (4). Increasing interest is arising on the bioactive fragments derived by PrP^C^ constitutive processing, not only for their physiological function but also because they appear to influence the course of prion and other neurodegenerative diseases (5, 6). α-cleavage is the main proteolytic event of PrP^C^, occurring between residues His_109_ and Lys_110_ (mouse sequence) in up to 50% of the molecules, depending on the cell type and tissue (7, 8, 9, 10, 11, 12). It produces the membrane-anchored C-terminal fragment C1 and the secreted N-terminal fragment N1. C1 is present in significant amount in healthy brain of several mammalian species and cell lines, and it is not a suitable substrate for conversion into PrP^Sc^, thereby *de facto* protecting against PrP^Sc^ propagation and disease progression (10, 11, 13, 14). A neuroprotective role has also been suggested for released N1 (15, 16, 17, 18). Despite some evidence suggesting that α-cleavage could be because of the action of one or more members of the ADAM (A Disintegrin And Metalloproteinase) family of enzymes, the identity of the responsible protease is still uncertain (9, 19, 20, 21). Analogous uncertainty remains about the cellular site of this event, as endosomal compartments, the late secretory pathway, and lipid raft domains of the membrane have all been suggested (19, 22, 23, 24). An alternative, but less prominent under physiological conditions, proteolytic event is β-cleavage, which occurs N terminal of the α-cleavage site at multiple nearby positions at the end of the octarepeat domain of PrP^C^, around amino acid 90 (10, 25, 26). It produces the released N-terminal fragment N2 and the membrane-bound C-terminal C2 fragment, that, in contrast to C1, seems to retain the ability to misfold (27). A C2-like fragment has been reported to be the main cleavage product of PrP^Sc^ in the brain of Creutzfeldt–Jakob disease patients (10, 25) and in prion-infected neuroblastoma cells (28), thus suggesting a pathophysiological relevance for β-cleavage. In physiological conditions, β-cleavage occurs at the cell surface because of a direct reactive oxygen species–mediated activity, which suggests an active role of PrP^C^ in the cellular protection against oxidative stress (26, 29, 30). In contrast, calpains and lysosomal proteases have been reported to mediate β-like cleavage during prion diseases (28, 31), thus indicating that this cleavage can be achieved by distinct mechanisms (6). A third physiological cleavage of PrP^C^ occurs at the very C terminus of the protein and results in the shedding of the almost full-length (FL) protein from the membrane, that is, shed PrP^C^ (7, 32, 33). To date, the exact site of PrP^C^ shedding has only been identified in mouse, between Gly_227_ and Arg_228_ (34, 35). The metalloprotease ADAM10 has so far been identified as the apparently sole sheddase of PrP^C^ (34, 36). Several hypotheses have been postulated on the function of PrP shedding. In physiological conditions, this extreme C-terminal cleavage is implicated in PrP^C^ homeostasis, but it has also been suggested that shed PrP^C^ holds some own independent functions as a soluble trophic factor acting on close or distant cells as well as a neuroprotective fragment in the extracellular space (19, 36). A conceivable dual role has been postulated for PrP shedding during prion diseases. While, on the one hand, it might confer protection against prion diseases as the reduction of PrP^C^ at the cellular surface would reduce the substrate for PrP^C^-to-PrP^Sc^ conversion, and shed PrP^C^ may impair this misfolding by binding to extracellular prion seeds (37, 38, 39, 40); on the other hand, it could facilitate prion spread and plaque formation by generating anchorless diffusible prions (32, 41, 42, 43, 44, 45, 46). Finally, a newly discovered endoproteolytic processing event of PrP^C^, named γ-cleavage, occurs at the C terminus of PrP^C^, supposedly between amino acids 176 and 200 (47, 48).

Although studied since decades for its essential role in prion replication (49) and neurotoxicity (50), PrP^C^ has acquired new interest in the latest years for its potential involvement in other neurodegenerative diseases, such as Alzheimer’s and Parkinson’s diseases. PrP^C^ has been reported to play a key role in the binding, internalization, and toxic signaling of amyloid-β, tau, and α-synuclein oligomers (51, 52). Increasing evidence supports the relevance of PrP^C^ proteoforms in health and disease, indicating that the proteolytic processing of PrP^C^ might influence the course of these neurodegenerative diseases. Indeed, α-cleavage and PrP^C^ shedding release N-terminal fragments able to bind and neutralize neurotoxic oligomers, while at the same time reducing FL-PrP^C^ on the cell membrane and, thus, its pathological consequences, such as PrP^Sc^ propagation in TSEs and oligomer-associated toxic signaling in other neurodegenerative diseases (6, 53). Owing to these emerging properties, PrP^C^ is increasingly considered a promising target for developing therapeutic strategies against TSEs and other neurodegenerative diseases (54, 55, 56), and targeting proteolytic cleavages of PrP^C^ may likewise become promising therapeutic options.

Despite the stated biological relevance of PrP^C^ proteolytic proteoforms, laboratory methods that allow a systematic and reliable identification and quantification of all these fragments are still lacking. Western blotting (WB) remains the most widely used technique for the identification of PrP^C^ cleavage products (25, 57, 58, 59), although other techniques have recently been employed (60, 61, 62). However, one of the most promising therapeutic targets, PrP^C^ shedding, produces proteoforms that remained mostly “undetectable” with the available methods for analyzing PrP^C^ in human or animal tissues and without cleavage site–specific antibodies. This is because of the fact that membrane-anchored and shed fragments have similar molecular weights (MWs) and, as the former are present in vast excess in most biological samples, the latter are therefore masked during readout. Building on previous knowledge on PrP^C^ processing and WB detection of PrP^C^ fragments, we aimed at exploring the potential of WB-based approaches able to fill this gap. Coupling PNGase F treatment of PrP^C^ with discriminative electrophoresis conditions and extensive epitope mapping, we developed an optimized and comprehensive WB assay able to detect all known PrP^C^ proteoforms and applied the new protocol to comparatively assess PrP^C^ endoproteolytic processing in the brain of bank voles, mice, and transgenic mice overexpressing ovine, bovine, or human PrP^C^.

## Results

### Identification of proteoforms derived from the endoproteolytic processing of PrP^C^

**Figure 1:**
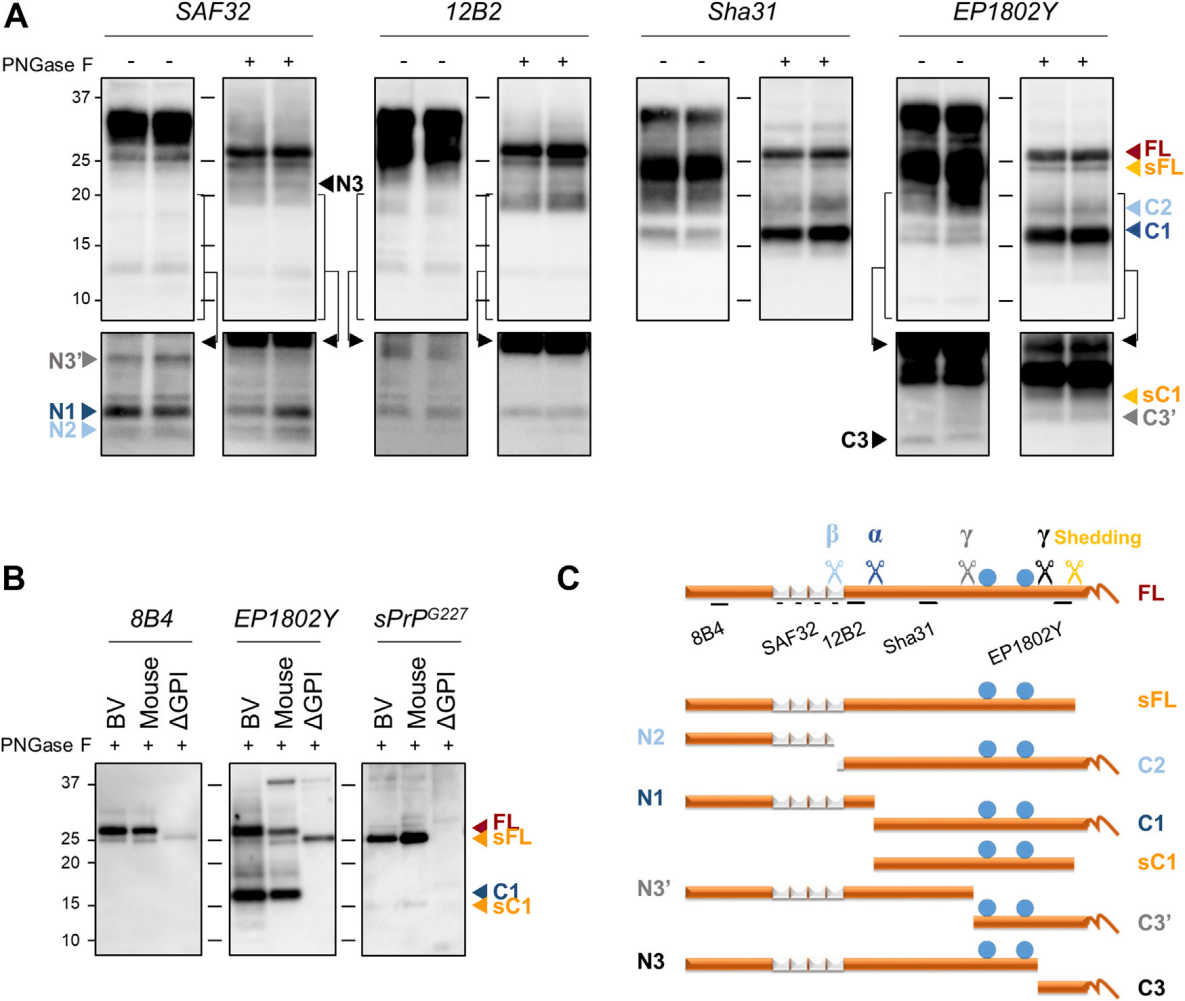
**Identification and schematic representation of bank vole PrP**^**C**^**proteoforms derived by endoproteolytic processing.***A*, representative Western blots of brain homogenates prepared from two healthy bank voles subjected to PNGase F treatment (+, *right blot* of each pair) or *left* untreated (−, *left blot* of each pair). Replica blots were probed with different antibodies (Abs), indicated at the top of each pair of blots. PrP^C^ proteoforms are indicated with colored *arrowheads*: *orange* is used for PrP^C^ proteoforms derived by shedding (shed full length [sFL] and shed C1 [sC1]), *blue* and *light blue* for PrP^C^ fragments derived by α- (N1/C1) and β-cleavages (N2/C2), and *gray* and *black* for PrP^C^ fragments derived by γ-cleavage (N3′/C3′ and C3/N3). A *red arrowhead* indicates full-length PrP^C^ (FL). Note that deglycosylation (+samples) was necessary to detect the glycosylated proteoforms of PrP^C^ (FL, C1, C2, sFL, sC1, C3′, and N3), whereas nonglycosylated fragments were more easily detected in untreated samples (N1, N2, N3′, and C3). A long exposure of the same blots (*black arrow*, the *bracket* indicates the portion of the blots shown after a longer exposure) was necessary for a clearer identification of less abundant PrP^C^ fragments. Tissue equivalents (TEs) loaded per lane were 0.2 mg for untreated (−) and 0.06 mg for PNGase F-treated (+) samples. The positions of molecular weight (MW) markers are indicated as *black lines* between “PNGase −“ and “+” blots, whereas the respective kilodaltons are reported on the left of the first blot. *B*, representative Western blots of PNGase F-treated brain homogenates from bank vole, WT mouse, and tgBVΔGPI. Replica blots were probed with the extreme N-terminal (8B4, first blot of the panel), extreme C-terminal (EP1802Y, second blot of the panel), and mouse shed PrP-specific (sPrP^G227^, third blot of the panel) antibodies. The latter (sPrP^G227^) detected a fragment with an apparent MW of approximately 25 kDa in both mouse and bank vole PNGase F, that is, sFL (*higher orange arrowhead*). EP1802Y and 8B4 detected the same fragment, few kilodaltons below FL (*red arrowhead*). The sPrP^G227^ antibody detected another fragment of approximately 14 kDa, that is, shed C1 (sC1), also recognized, few kilodaltons below C1 (*blue arrowhead*), by EP1802Y (*lower orange arrowhead*). TE loaded per lane were 0.06 mg for bank vole and WT mouse samples and 0.12 mg for tgBVΔGPI. The positions of MW markers are indicated as *black lines* between the blots, whereas the respective kilodaltons are reported on the left of the first one. *C*, linear representation of PrP^C^ (23–231) showing (i) the octarepeat region (repeated *white boxes*); (ii) the N-glycosylation sites (*blue spheres*, amino acids 181 and 197); (iii) the GPI-anchor (*curved line*, extreme C terminus); (iv) epitopes of pan-PrP antibodies used herein. *Colored scissors* indicate the main cleavage events and the respective cleavage products reported below. β-cleavage (*light blue scissors*) occurs at multiple sites at the end of the octarepeat domain of PrP^C^, around amino acid 90, and produces the soluble N-terminal N2 fragment of ∼10 kDa and the membrane-bound C-terminal fragment C2 (∼18 kDa). The major cleavage event, termed α-cleavage (*blue scissors*), occurs between residues H109 and K110 and produces the soluble N-terminal fragment N1 (∼12 kDa) and the ∼16 kDa C-terminal membrane anchored fragment C1. Shedding (*orange scissors*) occurs at the extreme C terminus of PrP^C^ resulting in the release of almost full-length PrP^C^, that is, sFL (∼25 kDa), or almost full-length C1 fragment, that is, sC1 (∼14 kDa), from the plasma membrane. Finally, γ-cleavage (*gray and black scissors*) occurs in the C-terminal region of PrP^C^, between amino acids 170 and 200. Our data indicate the presence of two “γ-cleavage-like sites,” one occurring N-terminal to the first N-glycosylation site (*gray scissors*) and producing the nonglycosylated soluble N-terminal fragment N3′ (∼17 kDa) and the ∼10 kDa glycosylated membrane-bound C3′, one occurring more C-terminally (*black scissors*), after the second N-glycosylation site, producing the ∼20 kDa glycosylated soluble N-terminal fragment N3 and the C-terminal nonglycosylated fragment C3 (∼7 kDa). PrP^C^, cellular prion protein.; sC1, shed C1; sFL, shed FL-PrP^C^.

Because of post-translational modifications and proteolytic cleavages, at steady state in brain tissue, what is generally referred to as “PrP^C^” is actually representing a complex ensemble of proteoforms, composed of membrane-anchored FL-PrP^C^ and several released and cell-associated proteolytic fragments. We first set up a protocol that allowed the discrimination and identification of all PrP^C^-derived fragments by established pan-PrP antibodies, including those proteoforms expected to have very similar migration in gel electrophoresis, such as FL-PrP^C^ and its shed counterpart, using bank vole brain homogenates. We found that, to allow for a high discrimination of PrP^C^ fragments, PrP^C^ disulphide bonds need to be kept strictly reduced during SDS-PAGE (see Experimental procedures section and Fig. S1). Moreover, it was necessary to inhibit the activity of endogenous proteases during all procedures in order to avoid the generation of nonspecific PrP-derived fragments that could hamper the proper identification of PrP^C^ proteoforms. We then selected a panel of widely used antibodies whose epitopes cover the whole sequence of PrP^C^ and are conserved in several species, taking into consideration the expected cleavage sites of PrP^C^. The presence of two C-terminal N-glycosylation sites in PrP^C^ further complicates the identification of each single proteoform, as FL-PrP^C^ and its C-terminal fragments migrate in gel electrophoresis as a plethora of partially overlapping diglycosylated, monoglycosylated, and nonglycosylated fragments (*e.g.*, see “− PNGase F” blots probed with Sha31 or EP1802Y in Fig. 1*A*). As shown in Figure 1*A*, enzymatic deglycosylation was key to the identification of these fragments as single discernible bands. Deglycosylation was also useful to identify unequivocally C-terminal and N-terminal proteoforms, as it induces an enrichment of natively glycosylated proteoforms relative to those that are natively nonglycosylated (see blots probed with SAF32 and 12B2 in Fig. 1*A*). Thus, we thoroughly compared untreated and deglycosylated samples with the whole set of antibodies, taking into consideration that deglycosylated samples were loaded approximately three to four times more diluted than untreated samples, so that nonglycosylated proteoforms are less prominent in deglycosylated than in untreated samples.

A detailed description of the PrP^C^ proteoforms detected in bank vole brain homogenates is reported later.

#### FL PrP^C^ and its α- and β-cleavage fragments in bank voles

Upon deglycosylation, FL-PrP^C^ was identified as a sharp band with an apparent MW of approximately 27 kDa that was recognized by the whole set of antibodies (Fig. 1*A*, *right blot of each panel*). In blots probed with C-terminal antibodies (Sha31 and EP1802Y), FL-PrP^C^ was accompanied by two main bands of ∼18 and ∼16 kDa, with the former also being detected with 12B2 (Fig. 1*A*). Based on their apparent MW, glycosylation status, and antibodies reactivity, these two fragments were identified as the less abundant β-cleavage C-terminal fragment C2 and the most abundant α-cleavage fragment C1, respectively (Fig. 1, *A* and *C*). Consistent with the reported multiple β-cleavage sites (resulting from different cleavage events in a region around amino acid 90), C2 appeared as a rather diffuse band, whereas C1 was detected as a sharp band. As expected, the N-terminal monoclonal antibodies (mAbs) SAF32 and 12B2 detected the N-terminal counterparts of these proteoforms. Antibody SAF32 detected two fragments with apparent MW of approximately 12 and 10 kDa, whereas mAb 12B2 mainly recognized only the upper 12 kDa band. A lower fragment was barely detected by 12B2 as an extremely faint band with an apparent MW corresponding to the MW of the upper portion of the 10 kDa band detected by SAF32. Based on these features, we identified the two fragments as N1 and N2, respectively, with N1↓C1 being located between the 12B2 and Sha31 epitopes and N2↓C2 multiple cleavage sites occurring mainly N-terminally to the 12B2 epitope _88_WGQGG_92_ (Fig. 1, *A* and *C*). In keeping with the relative abundance of C1 and C2, N1 was much more abundant than N2, despite the amount observed for both N-terminal fragments was much less than that of their C-terminal counterparts (Fig. 1*A*, see “+ PNGase F” blots probed with mAbs SAF32 and EP1802Y and compare N-terminal and C-terminal proteoforms with FL-PrP^C^).

#### PrP^C^ shedding in bank voles

Another sharp band running ∼2 kDa below the FL-PrP^C^ band was detected in PNGase F-treated samples by the whole set of antibodies (Fig. 1*A*, “+ PNGase F” blot of each panel). Its apparent MW and antibodies reactivity suggested that this fragment could be shed FL-PrP^C^ (sFL). To test this hypothesis, we used a recently generated shed PrP^C^-specific antibody (sPrP^G227^; note that this antibody, based on a previous publication (34), has initially been named “sPrP^G228^” (63), yet the relevant glycine in the murine sequence is actually positioned at 227). This antibody was produced using the mouse recombinant peptide NH_2_-C-QAYYDG_227_-COOH as an immunogen, with G_227_-COOH being the newly formed C terminus after shedding by ADAM10, so that sPrPG^227^ exclusively detects shed PrP of some rodents (63). Because of the presence of the same glycine in the bank vole PrP sequence (Gly_228_ in bank vole, Table 1), we tested if sPrP^G227^ would recognize bank vole shed PrP^C^, too. As shown in Figure 1*B*, the sPrP^G227^ antibody detected a fragment with an apparent MW of ∼25 kDa in both mouse and bank vole PNGase F-treated brain homogenates but not in tgBVΔGPI that express an artificial version of vole shed PrP^C^ with Ser_231_ C-terminal truncation. The same fragment, running just slightly below FL-PrP^C^, was detected by the extreme C-terminal antibody EP1802Y and by the extreme N-terminal antibody 8B4 (Fig. 1, *B* and *C*; as expected, these mAbs also detected anchorless PrP^C^ in tgBVΔGPI, though with different sensitivity). We thus concluded that the fragment detected by all antibodies at ∼25 kDa is indeed sFL. Interestingly, our electrophoresis conditions proved to efficiently discriminate PrP^C^ fragments differing in very few residues, as shown by the different apparent MWs shown by PrP(23–231) in tgBVΔGPI and mouse shed PrP(23–227) (compare lanes 2 and 3 in Fig. 1*B*).

**Table 1:** Analysis of the C-terminal PrP residues in different mammalian species

Species	C-terminal PrP residues^a^		
Mouse^b^	208	VVEQMCVTQYQKESQAYYD**G**RRSS	231
Bank vole	209	VVEQMCVTQYQKESQAYYEGRSSR	232
Sheep	212	VVEQMCITQYQRESQAYYQRGASV	235
Bovine	220	VVEQMCITQYQRESQAYYQRGASV	243
Human	209	VVEQMCITQYERESQAYYQRGSSM	232

a The C-terminal region of mouse PrP spanning residues 208 to 231 has been used as query for the sequence alignment analysis.

b The glycine identified in bold represents the newly formed C-terminal end of PrP after ADAM10 cleavage in mouse (Gly_227_), whereas residues underlined represent the critical epitope region of the antibody EP1802Y (70).

We also noticed that antibody sPrP^G227^ detected relatively less shed PrP^C^ signal in bank vole than in mouse brain. However, we could not discriminate at this stage if this indicates a lower PrP^C^ shedding in bank vole or merely reflects a lower affinity of the polyclonal sPrP^G227^ antibody for bank vole shed PrP^C^ because of the D/E substitution at residue 226 (mouse sequence, Table 1).

Antibody sPrP^G227^ also weakly detected another shed PrP^C^ fragment with an apparent MW of ∼14 kDa, that is, shed C1 (sC1) (Fig. 1*B*), that was also detected by the C-terminal mAbs Sha31 and EP1802Y as a weak band just below C1 (Fig. 1, *A* and *C*). In line with what was observed earlier (63), sC1 was much less abundant than sFL, despite the amount of C1 present in a healthy brain exceeding that of FL-PrP^C^ (see Sha31 and EP1802Y blots in Fig. 1*A*), thus supporting previous evidence that the preferred substrate of ADAM10 is FL-PrP^C^ (63).

Finally, the detection of shed PrP^C^ in bank vole by the anti-shed antibody sPrP^G227^ demonstrates for the first time that PrP shedding (likely mediated by ADAM10) occurs at the Gly_228_–Arg_229_ peptide bond in these rodents widely used in prion research.

#### γ-cleavage of PrP^C^ in bank voles

A novel minor endoproteolytic event within the C-terminal region of PrP^C^, designed as γ-cleavage, has recently been identified in mouse and human brain samples (48). According to Lewis *et al.*, γ-cleavage preferentially occurs on nonglycosylated PrP^C^ between residues 176 and 200, resulting in the production of a GPI-anchored nonglycosylated C3 fragment with an apparent MW of 6-7 kDa exclusively detected by the extreme C-terminal antibody EP1802Y. The N-terminal counterpart of this fragment, N3, has been identified as a fragment with an apparent MW of ∼20 kDa, detected by mAb SAF32. The authors also reported another C-terminal fragment, C3′, again exclusively detected by EP1802Y few kilodaltons below C1 upon deglycosylation, which they proposed as a C3 precursor or an intermediate, that is, C3′. We thus aimed at verifying the presence of γ-cleavage in bank vole brain homogenates with our “PNGase F −/+” method. Indeed, EP1802Y detected a nonglycosylated 6-7 kDa fragment consistent with C3 (Fig. 1*A*, left blot probed with EP1802Y). Two other fragments of ∼13-14 kDa and ∼11-12 kDa were detected by EP1802Y exclusively in “+ PNGase F” samples (Fig. 1*A*, right blot probed with EP1802Y). We identified the former, also detected with mAb Sha31, as sC1 (see previous section), whereas the latter was consistent with C3′. However, the identification of C3′ only upon PNGase F treatment led us to conclude that it is a glycosylated fragment. The C-terminal antibody SAF84 did not detect C3 and C3′, thus confirming their cleavage sites to occur C-terminally to the epitope _160_VYYRPVDQY_169_ (bank vole sequence) (Fig. S2).

Based on their different glycosylation status, epitope mapping, and apparent MW, the production of C3 and C3′ could be because of the presence of multiple γ-cleavage-like sites, one taking place C-terminally to residue 198 and producing the nonglycosylated 6 to 7 kDa C3, one occurring between residues 170 and 181, and determining the glycosylated 11-12 kDa C3′. If so, two N-terminal counterparts should be detected: a glycosylated fragment with an apparent MW of ∼20 kDa for C3 and a ∼15-17 kDa nonglycosylated fragment for C3′. The N-terminal antibodies 8B4 and SAF32 detected a PrP^C^ fragment of ∼17 kDa that was not glycosylated as it was not enriched following deglycosylation (Fig. 1*A*, left blot probed with SAF32 and Fig. S3). Although a similar fragment has been reported by Lewis *et al.* (48) as N3, based on its apparent MW and lack of N-linked glycans observed here, we propose that it represents N3′, that is, the N-terminal counterpart of the glycosylated fragment C3′. Given the characteristics of C3, N3 was instead supposed to be detected in PNGase F-treated brain homogenates just few kilodaltons below sFL and above C2. A fragment of ∼19-20 kDa was indeed detected in PNGase F-treated samples by mAbs SAF32 and 8B4 (Fig. 1*A*, right blot probed with SAF32 and Fig. S3). However, 12B2 and Sha31 did not unequivocally detect this fragment, as it was likely masked by the more abundant C2, thus preventing its indisputable identification as N3.

These data confirm the presence of γ-cleavage in bank voles and suggest the existence of two “γ-cleavage-like sites,” one N-terminal (C3′ + N3′) and one C-terminal (C3 + N3) to the two N-glycosylation sites of PrP^C^ (Fig. 1*C*).

### Endoproteolytic processing of PrP^C^ in wild type mice

**Figure 2:**
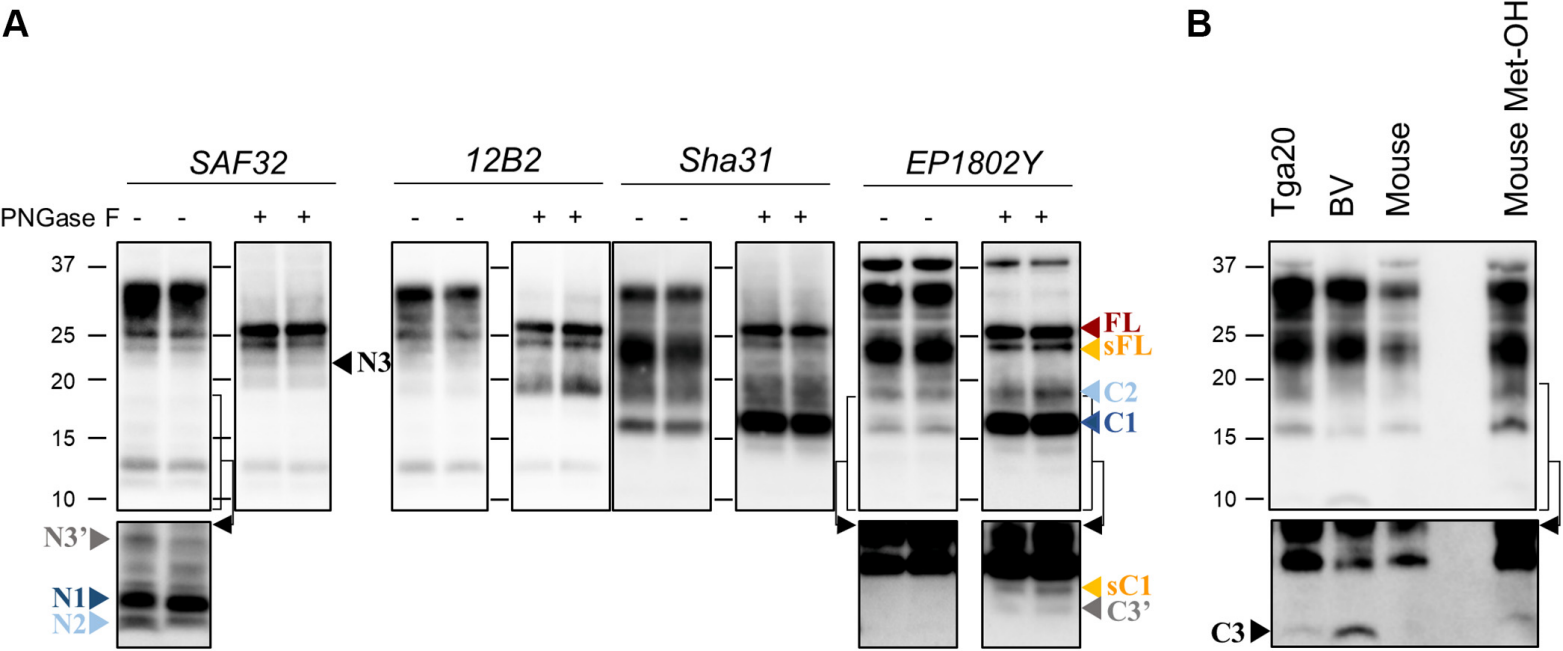
**Identification of mouse PrP**^**C**^**proteoforms derived by endoproteolytic processing.***A*, representative Western blots of brain homogenates prepared from two WT mice subjected to PNGase F treatment (+, *right blot* of each pair of blots) or untreated (−, *left blot* of each pair). Replica blots were probed with different antibodies (Abs), indicated at the top of each pair. PrP^C^ proteoforms are indicated with *arrowhead* colored differently for different proteolytic events: *orange* is used for shedding (sFL and sC1), *blue* and *light blue* for α- (N1/C1) and β-cleavages (N2/C2), and *gray* and *black* for γ-cleavages (N3/C3 and N3′/C3′). A *red arrowhead* indicates FL-PrP^C^. Note that in untreated samples, C3 was not detected (see blot probed with Ab EP1802Y, “−“ samples), whereas its N-terminal counterpart was detected in PNGase F-treated samples (see blot probed with SAF32, “+” samples). A long exposure (*black arrow*, the *bracket* indicates the portion of the blot shown after to a longer exposure) was necessary for a clearer identification of less abundant PrP^C^ fragments. The positions of molecular weight (MW) markers are indicated as *black lines* between “PNGase −” and “+” blots, whereas the respective kilodaltons are reported on the left of the first blot. *B*, representative Western blot of untreated brain homogenates from a bank vole, a WT and a tga20 mouse analyzed with EP1802Y. WT mouse sample was also analyzed after methanol precipitation (Met-OH) to increase the amount of sample loaded (last lane of the blot). C3 was detected in bank vole and tga20 mouse brain, whereas methanol precipitation was necessary for its detection in the WT mouse sample. Tissue equivalents (TEs) loaded per lane were 0.2 and 0.06 mg for untreated (−) and PNGase F-treated (+) samples, whereas 1 mg was used for the methanol-precipitated sample. A long exposure (*black arrow*, the *bracket* indicates the portion of the blot shown after to a longer exposure) was necessary for a clearer identification of less abundant PrP^C^ fragments. The positions of MW markers (and the respective kilodaltons) are reported on the *left*. PrP^C^, cellular prion protein; sC1, shed C1; sFL, shed FL-PrP^C^.

Having established a method that allows to reliably identify all relevant PrP^C^ proteoforms, we next aimed at comparing the endoproteolytic processing of PrP^C^ of bank voles and wild type mice, two rodent models that show different susceptibility to prion diseases (64, 65, 66, 67, 68, 69, 70, 71). We first aimed to confirm antibodies reactivity in mouse brain and found that all antibodies but EP1802Y equally detected vole and mouse PrPs (Fig. S4). Sequence alignment showed that the critical epitope-binding region for EP1802Y was conserved in both species (72), but substitutions were observed at residues 226, 229, and 231 (Table 1). The preferable detection of bank vole PrP^C^ by EP1802Y could be tentatively ascribed to these substitutions, especially to the D226E one that flanks the critical Ab/PrP-binding region, which could modulate binding activity. As shown in Figure 2, the method could be efficiently used also in the analysis of wild type mouse PrP^C^ endoproteolytic processing. The main PrP^C^ proteoforms detected in mouse were strikingly similar to those of bank vole in terms of number, apparent MW, and chemiluminescent signal (compare Figs. 1*A* and 2*A*). C3, instead, was not detected in mouse brain homogenates, which might be because of a low abundance in combination with the lower sensitivity of mAb EP1802Y to mouse PrP^C^. Indeed, C3 can be detected by loading a higher amount of sample or by analyzing brain homogenates from tga20 mice that overexpress mouse PrP^C^, although apparently still at lower levels than in voles (Fig. 2*B*). The unequal detection of mouse and bank vole PrP by EP1802Y, however, did not allow for drawing definite conclusions on this issue. Of note, mouse γ-cleavage processing of PrP^C^ matched our previous data in bank voles, as, together with C3, the C-terminal fragment C3′ (Fig. 2*A*, see right blot probed with EP1802Y) and the two N-terminal fragments N3 and N3′ (Fig. 2*A*, see blots probed with SAF32) were detected.

### Endoproteolytic processing of PrP^C^ in transgenic mice

**Figure 3:**
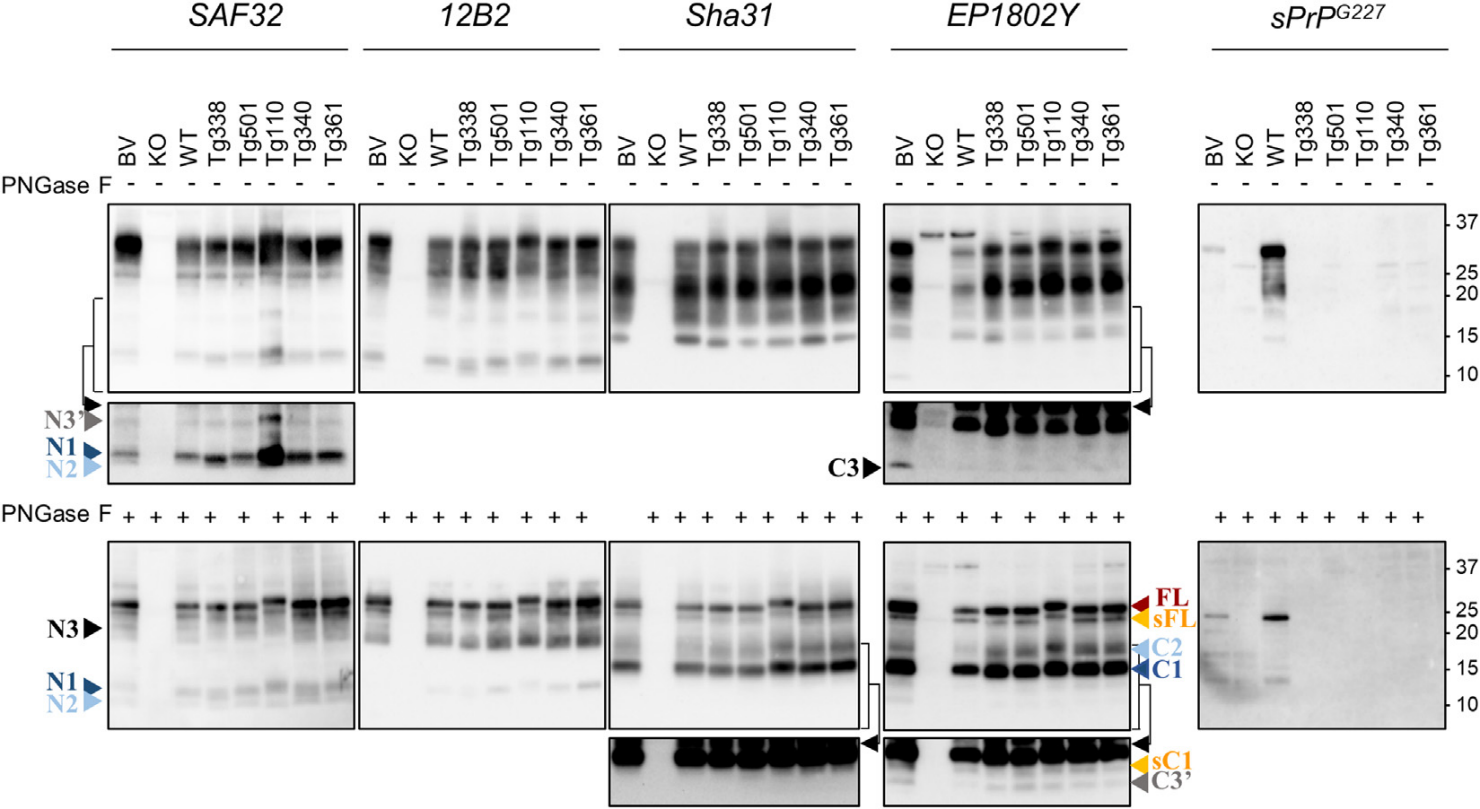
**Comparison of PrP**^**C**^**proteoforms present in the brain ofwild typeand transgenic rodent models.** Representative western blots of brain homogenates prepared from bank vole, PrP-KO mouse (KO), wild type mouse (WT), tg338 (expressing sheep-VRQ PrP^C^), tg501 (expressing sheep/goat-ARQ PrP^C^), tg110 (expressing bovine PrP^C^), tg340 and tg361 (expressing human M129 and human V129 PrP^C^, respectively). All samples were left untreated (“−,” *upper* blots of the panel) or subjected to PNGase F treatment (“+,” *lower blots* of the panel). Replica blots were probed with different antibodies, indicated at the *top* of each pair. PrP^C^ proteoforms are indicated with *arrowhead* colored differently for different proteolytic events: *orange* is used for shedding (sFL and sC1), *blue* and *light blue* for α- (N1/C1) and β-cleavages (N2/C2), and *gray* and *black* for γ-cleavages (N3/C3 and N3′/C3′). A *red arrowhead* indicates FL-PrP^C^. Note that the overall PrP^C^ proteoform pattern is very similar among the rodent models, apart from the apparent molecular weight (MW) of FL, sFL, C2, N1, and N3, which is higher in tg110 than in other models because of an extra octarepeat in bovine PrP^C^. As expected because of sequence alterations, sFL was detected in none of the transgenic mouse lines by the mouse sPrP-specific antibody (*upper* and *lower blots* on the *right*, *orange arrowhead*). Tissue equivalents (TEs) loaded per lane were 0.2 and 0.06 mg for untreated (−) and PNGase F-treated (+) samples. The positions of MW markers (and the respective kilodaltons) are reported on the *right*. A long exposure (*black arrow*, the *bracket* indicates the portion of the blot shown after a longer exposure) was necessary for a clearer identification of less abundant PrP^C^ fragments. PrP^C^, cellular prion protein; sC1, shed C1; sFL, shed FL-PrP^C^.

Transgenic mouse lines overexpressing PrP from different species on a PrP^−/−^ background are pivotal in the study of prion strains, pathological differences, and species barriers (73). Still, it has not been systematically investigated if sequence variations, overexpression levels, or the heterogeneity between exogenous/transgenic PrP^C^ and the murine cellular environment in which it is processed could have any impact on the physiological endoproteolytic processing of PrP^C^ and its putative disease-modifying potential in these models. We thus analyzed a panel of transgenic mice expressing sheep-VRQ (tg338), goat/sheep-ARQ (tg501), bovine (tg110), and human-M129 and human-V129 (tg340 and tg361, respectively) PrP^C^ to explore the PrP^C^ proteoforms present in these models and to compare them with those of wild type models. A first analysis showed that PrP^C^ proteoforms were similarly recognized in all the models by the whole set of antibodies (Fig. 3), EP1802Y included. Indeed, multiple alignment showed that the critical epitope-binding region was conserved among all sequences, despite the presence of some substitutions nearby the conserved epitope (Table 1). Based on the combined analysis of sequence alignment and WB detection of the entire set of PrPs analyzed, our results suggest that D226E represents the only detrimental substitution outside the critical EP1802Y-binding region. As shown in Figure 3, no major differences were detected among the wild type models and the tg mice in terms of number, apparent MW, and chemiluminescent signal of the most abundant PrP^C^ fragments, with the exception of the apparent MW of some PrP^C^ proteoforms in tg110 (Figs. 3 and S5). Indeed, as expected, the presence of an extra octarepeat region in bovine PrP^C^, FL-PrP^C^, sFL, and the N-terminal fragments N1 and N3 showed an apparent MW slightly higher in tg110 than in the other mouse lines (Fig. 3, *lower panel*). A slight increase of the apparent MW was also visible for C2, but not for N2 and C1, indicating that, in this model, β-cleavage occurs N-terminal to the extra GGWGQPHG repeat, which is thus retained in C2. Among the less abundant fragments, C3 was detected in all transgenic mouse lines at lower levels than in bank voles (Fig. 3, upper blot probed with EP1802Y and Fig. S6), whereas N3 was not always clearly distinguishable (Fig. 3, lower blot probed with SAF32); in contrast, C3′ and N3′ were distinctly identified in all tg mouse lines, better than in wild type mouse and similarly to bank vole (Fig. 3, lower blot probed with EP1802Y). Despite these data suggest some variation of PrP^C^ γ-cleavage processing between bank vole, wild type mouse, and transgenic mouse lines, the generally low abundance of these fragments prevented a more detailed analysis. Finally, sFL was consistently detected as a distinct band in all tg mouse lines, despite the fact that sheep, bovine, and human PrPs share the conserved sequence _227_QRG_229_ that differs from the mouse counterpart (Table 1). Fittingly, the sPrP^G227^ antibody did not detect shed PrP^C^ in any of the tg mouse lines (Fig. 3), thus confirming that none of the transgenically expressed PrPs analyzed here share the murine shedding site. These findings also indicate that mouse ADAM10 recognizes heterologous shedding sites in exogenous PrPs and that, in the tg mouse lines, murine ADAM10 operates by preserving the exogenous PrP^C^ cleavage sites of those respective species.

### Quantitative assessment of endoproteolytic processing of PrP^C^ in wild type and transgenic rodents

We then evaluated if our method could be exploited for quantitative assessment of the relative amounts of the most abundant and, likely, physiologically, most relevant PrP^C^ proteoforms, that is, N1, N2, C1, C2, sFL, and FL-PrP^C^. In principle, these proteoforms could be easily quantified relative to FL-PrP^C^ in PNGase-treated samples using two antibodies: SAF32 for N1 and N2 and EP1802Y for C1, C2, and sFL. Importantly, EP1802Y is ideal for the quantification of C2 and sFL, as it does not detect any N-terminal fragment that might overlap with C2, and it is not affected by a nonspecific band at ∼25 kDa detected by the antimouse secondary antibody (Fig. S7). However, we noticed that EP1802Y has an intrinsically lower affinity for C1 than for the other PrP^C^ proteoforms, thus preventing a reliable assessment of the relative abundance of C1 across the animal models (Fig. S4). Thus, we introduced a third antibody, Sha31, for the assessment of C1.

**Figure 4:**
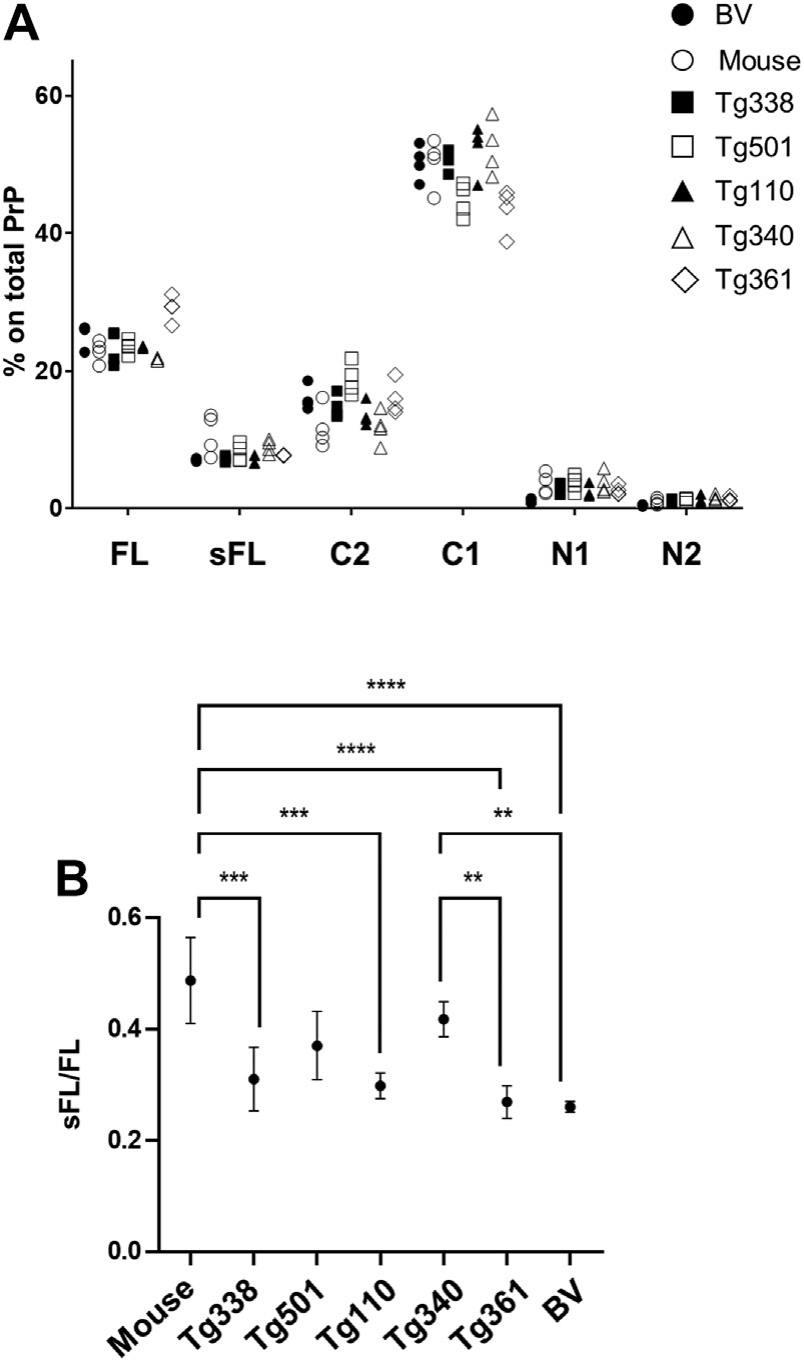
**Quantitative assessment of the most abundant PrP**^**C**^**proteoforms inwild typeand tg rodent models.***A*, graph depicting the relative abundance of FL-PrP^C^, sFL, C1, C2, N1, and N2 in the brain of wild type mice, bank voles, as well as tg338, tg501, tg110, tg340, and tg340 mice. The data are derived combining the quantification of proteoforms relative to FL-PrP^C^ detected with SAF32 (for the quantification N1 and N2), Sha31 (for C1), and EP1802Y (for sFL and C2), as described in the Experimental procedures section. Each point represents the value of a single individual animal, and the value of four individuals for rodent model are reported (see also Table 2 for mean ± SD of each rodent model). *B*, graph depicting the sFL/FL ratio in the set of rodent models analyzed. The ratio was determined by calculating the chemiluminescence signal of FL-PrP^C^ (FL) and its shed counterpart (sFL) detected by mAb EP1802Y in n = 4 individuals per model. The analysis was repeated three times, and the mean value ± SD for each rodent model are shown. One-way ANOVA showed significant variability among the models (*F* = 12.81; *p* < 0.0001; *R*^2^ = 0.785). ∗∗, ∗∗∗, and ∗∗∗∗ indicate *p* < 0.01, *p* < 0.001, and *p* < 0.0001, respectively, in pairwise comparisons (Tukey's multiple test). PrP^C^, cellular prion protein; sC1, shed C1; sFL, shed FL-PrP^C^.

We found remarkably similar PrP^C^ proteolytic processing patterns among animal models, which all showed the same rank order of PrP^C^ proteoforms, that is, C1 > FL > C2 > sFL > N1 > N2 (Fig. 4*A* and Table 2). Interestingly, C1 was by far the most abundant proteoform in all models, accounting for 45 to 52% of total PrP^C^, that is, two to three times more than FL-PrP^C^ (22–29%) and C2 (12–19%). Surprisingly, shed PrP^C^ (sFL), the fragment typically masked in WB analyses of brain samples and often thought to represent a rather minor fraction, also represented a substantial quote of total PrP^C^, accounting for 7 to 10% of total PrP^C^, that is, only twofold to fourfold less than membrane-anchored FL-PrP^C^ (Table 2). Of note, the N-terminal fragments N1 and N2 were much less abundant than their C-terminal counterparts, which may in part be because of the described low biostability of these fragments upon release (74).

**Table 2:** Relative abundance of PrP^C^ proteoforms in the brain of the rodent models analyzed

Line	Percent of PrP^C^ proteoforms on total PrP^C^ (mean ± SD)					
	FL-PrP	sFL	C2	C1	N1	N2
Bank vole	25.05 ± 1.35	6.65 ± 0.69	16.88 ± 1.08	49.94 ± 0.39	1.08 ± 0.37	0.39 ± 0.12
C57Black	22.81 ± 1.54	10.74 ± 2.95	11.74 ± 3.06	50.22 ± 3.60	3.53 ± 1.54	0.96 ± 0.45
Tg338	23.31 ± 2.42	7.19 ± 0.41	14.71 ± 1.68	50.65 ± 1.54	2.99 ± 0.79	1.15 ±0.20
Tg501	23.45 ± 0.99	8.08 ± 1.26	18.81 ± 2.31	44.78 ± 2.42	3.64 ± 1.16	1.23 ± 0.29
Tg110	23.38 ± 0.17	7.14 ± 0.64	13.57 ± 1.67	52.29 ± 3.63	2.42 ± 0.89	1.20 ± 0.58
Tg340	21.65 ± 0.21	9.01 ± 0.98	11.76 ± 2.35	52.36 ± 3.99	3.72 ± 1.53	1.50 ± 0.43
Tg361	29.05 ± 1.87	7.70 ± 0.07	15.97 ± 2.43	43.39 ± 3.23	2.59 ± 0.68	1.30 ± 0.33

Despite the similar overall patterns observed, the data also suggested some differences in the relative abundance of PrP^C^ fragments among the models (Table 2). Unfortunately, these quantitative data were not easily amenable to statistical assessment because of the inherent nature of data derived by using three different antibodies, which could have an impact on the precision and homogeneity of quantification particularly when comparing different sequences. We argued that it could be possible to obtain statistically assessable values by evaluating the relative quantification of pairs of PrP^C^ fragments detected on the same blot by a single antibody. We focused on the quantification of shed PrP^C^, by evaluating the sFL/FL ratio, for the following reasons: (i) shed PrP^C^ seemed to vary between the wild type models of mouse and bank vole (Table 2); (ii) these two proteoforms have similar chemical composition and apparent MW, since they only differ for few amino acids plus the presence/absence of the GPI anchor, and they can both be detected by the same antibody (thus bypassing the potential technical distortions); and (iii) such quantification has not been possible so far with other methods. The quantitative analysis with mAb EP1802Y (Fig. 4*B*) confirmed that the two wild type rodent models significantly differed in their relative PrP^C^ shedding, with wild type mice showing the highest amount of shed PrP^C^ (with an sFL/FL ratio of 0.50 ± 0.11) and bank voles the lowest (0.26 ± 0.02). One-way ANOVA showed a significant variability among the models (*F* = 12.81; *p* < 0.0001; *R*^2^ = 0.785), with highly significant differences between wild type mice and bank voles, as well as between wild type and some transgenic mouse lines, such as tg338, tg110, and tg361 (Fig. 4*B*). The bank vole sFL/FL ratio was also significantly different from tg340 (Fig. 4*B*), which was characterized by a particularly high sFL/FL ratio (0.42 ± 0.05). Interestingly, tg340 was significantly different from tg361 (Fig. 4*B*), from which it differs only for the polymorphism Met/Val at position 129. Quantifications of sFL/FL with Sha31, 12B2, and SAF32 confirmed these variations (Fig. S8), although showing less marked differences probably because of the presence of the unspecific band of 25 kDa detected by their secondary antibody (Fig. S7). Overall, the sFL/FL ratio in wild type mice was significantly different from bank vole and tg361 with all mAbs tested, and from tg338 and tg110 with two of four mAbs, whereas tg340 was also significantly different from bank vole and tg361 with three of four mAbs (Fig. S8).

As the two wild type rodents, that is, mouse and bank vole, showed significantly different amount of shed PrP^C^, we argued that it could be possible to further evaluate the role of PrP primary sequence on PrP^C^ shedding levels by analyzing the sFL/FL ratio of a tg mouse line expressing bank vole PrP (tg407). The quantitative analysis with mAbs EP1802Y and Sha31 showed comparable levels of shed PrP^C^ between bank voles and the transgenic mice expressing bank vole PrP and confirmed significant variability between the two models and wild type mice (Fig. S9).

## Discussion

An increasing body of evidence indicates that PrP^C^ is implicated in diverse pathophysiological aspects of prion and other more common neurodegenerative diseases, including Alzheimer’s and Parkinson’s diseases (75, 76, 77). Truncated PrP^C^ proteoforms generated by conserved and constitutive endoproteolytic processing events have been suggested to be pivotal as well, as they show different propensity to misfolding and binding of neurotoxic ligands (6). Despite the importance of PrP^C^ as a pharmacodynamic biomarker and the amount of studies focusing on proteolytic processing of PrP^C^, recently developed methods allow to determine the overall concentration of PrP^C^ (60, 61, 78) or that of its main proteolytic fragments C1 and C2 (62) but fail to give a comprehensive representation of all relevant PrP^C^ proteoforms and of their possible disease-associated alterations. Most importantly, none of the available methods is able to clearly discriminate shed PrP^C^, which may be one of the most promising prion therapeutics targets (37, 79), from GPI-anchored FL-PrP^C^. Although the recent generation of cleavage-site specific antibodies exclusively detecting shed PrP^C^ enabled studies investigating ADAM10-mediated cleavage of PrP^C^ (37, 63), the quantitative assessment of this cleavage (*e.g.*, sFL in relation to FL-PrP^C^ or total PrP^C^ within one sample) remained problematic. In this study, we show that all PrP^C^ proteoforms can be identified by using an optimized WB assay that combines (i) deglycosylation treatment, which simplifies the visualization of PrP^C^ fragments, (ii) discriminative electrophoresis conditions, which allow the effective separation of PrP^C^ bands of similar MW, and (iii) extensive epitope mapping, which was key for the identification of the different proteoforms. With this approach, we obtained the maximum information about PrP^C^ constitutive processing and the abundance of respective fragments in a complex biological sample, essentially being able to identify 11 PrP^C^ proteoforms in brain homogenates of mice and voles, that is, five GPI-anchored/C-terminal (FL, C1, C2, C3, and C3′), four released N-terminal (N1, N2, N3, and N3′), and two C-terminally shed (sFL and sC1) proteoforms. Along with a comprehensive description of the endogenous PrP^C^ cleavages, the method also allowed to quantify the most abundant PrP^C^ proteoforms. We report that the relative amount of PrP^C^ proteoforms in the brain is remarkably similar in the rodent models investigated herein, with a rank order of abundance of C1 > FL > C2 > sFL > N1 > N2. As we compared different species, that is, mouse *versus* bank voles, as well as wild type *versus* transgenic mice overexpressing PrP^C^ from different species, these findings imply that variations in PrP^C^ sequence or level of expression, different genetic backgrounds, or the heterogeneity between heterologous PrP^C^ and the mouse cellular proteolytic machinery do not have a major impact on PrP^C^ endoproteolytic processing. Such similarity in PrP^C^ processing across species is in agreement with the overall conservation of PrP primary structures and its proteolytic cleavage sites, as for α- and β-cleavage (80). Indeed, α-cleavage, whose cleavage site itself is extremely conserved (_109_HK_110_), is located within the most highly conserved PrP region (100–120), whereas β-cleavage occurs in a region of PrP whose predominant part is a repeating structure (mostly glycine and proline) and in which various peptides are conserved across species. Altogether, these data support the high conservation not only of mature FL-PrP^C^ but also of its cleavage events and therefore call for further studies investigating conceivable intrinsic functions of the resulting fragments.

Of note, GPI-anchored proteoforms were the most abundant ones in brain, with the sum of FL, C1, and C2 representing around 80% of total PrP^C^. In line with what was reported for cell lines and tissues (8, 10, 14, 57), C1 was by far the most abundant PrP^C^ proteoform in the brain, representing up to 50% of total brain PrP^C^, thereby being approximately two times more abundant than FL-PrP^C^. In sharp contrast, the released N-terminal products of α- and β-cleavages were much less represented than their C-terminal counterparts at steady state, thus suggesting that their diffusion in the extracellular space, drainage *via* body fluids, and/or a putative lower biostability might account for this difference. However, we found that sFL, yet another released fragment, represents an appreciable proportion of total PrP^C^, as it accounted for 7 to 10% of total PrP^C^, thus suggesting that the ADAM10-mediated shedding is constitutively highly active in the brain and that shed PrP^C^, which still harbors the C-terminal globular domain, might be more stable than N1/N2 fragments that miss that part. Both aspects may hint at relevant physiological roles played by this proteoform.

A surprising finding of this study was that only 20 to 25% of total brain PrP^C^ is present at the plasma membrane as FL-PrP^C^. Moreover, the relative amount of the main proteoforms, including the amount of GPI-anchored FL-PrP^C^, was very similar among the animal models investigated, notwithstanding the overexpression of heterologous PrP^C^ in transgenic mouse lines. These findings have several implications for a better understanding of the pathophysiological consequences of PrP^C^ proteolytic processing. Indeed, they imply that these processing steps are key to ensure the homeostatic balance of brain PrP^C^ proteoforms, strictly maintaining FL-anchored PrP^C^ close to a “safe” threshold on the one hand, while producing controlled amounts of the proteolytic proteoforms, with physiological and potentially neuroprotective roles, on the other hand. It would be interesting to determine the PrP^C^ proteoform profiles in tissues other than the brain, as this could provide insights into the function of the different proteoforms of the rather widely expressed PrP^C^. For example, the unexpectedly high C1 concentration in brain could be related to the reported neuroprotective role of this proteoform (11) and of its corresponding N1 fragment (17, 18), which could suggest lower rates of α-cleavage and, hence, amounts of C1 outside the brain. Interestingly, these data also imply that pathophysiological conditions affecting the proteolytic processing of PrP^C^ could lead to an increase of the misfolding-prone GPI-anchored FL-PrP^C^, potentially leading to pathological consequences. In this context, it would be interesting to determine if aging, the main risk factor for sporadic prion diseases, affects the PrP^C^ proteoform composition in the brain.

The most important new feature of the analytical method we have developed resides in the ability to detect sFL simultaneously to FL-PrP^C^ and, hence, to assess proteolytic shedding by ADAM10. A fundamental step in this direction is represented by the development of an antibody that specifically detects shed PrP, that is, PrP fragments ending with Gly_227_ exposed at C terminus after ADAM10 cleavage in mice and rats (63), which has been pivotal for the confirmation of our WB method. However, the sPrP^G227^ antibody only allows an indirect comparison between shed PrP^C^ and the GPI-anchored proteoforms, and it is specific for murine/rat shed PrP^C^. Studies on PrP^C^ shedding in other species are not possible with that tool because of altered cleavage site sequences. With our optimized method, we are now able to reliably and sensitively detect shed PrP^C^ and discriminate it from FL-PrP^C^ regardless of the pan-PrP antibody used and the PrP^C^ sequence analyzed. We could thus directly compare sFL and all the other GPI-anchored proteoforms independently on the specific cleavage site, *de facto* widening the plethora of species in which shed PrP^C^ can be analyzed and enabling to quantitatively assess PrP^C^ shedding and its relation to other PrP^C^ fragments or total PrP^C^ (including potential disease-related changes in this ratio). As the need to detect sFL may become increasingly crucial in prion and other neurodegenerative diseases because of its biomarker and therapeutic potential (37, 81, 82, 83), we anticipate that the method proposed herein will allow to gain further insights into functions and implications of shed PrP^C^ and the ADAM10-mediated shedding process. The detection of shed PrP^C^ in transgenic mouse lines overexpressing heterologous PrPs clearly represents the first of these insights, as this indicates that a correct shedding occurs despite ADAM10/PrP^C^ species heterogeneity, as mouse ADAM10 cleaves sheep, human, and bovine PrPs although they lack the respective mouse _227_GR_228_ cleavage site. These findings suggest that amino acid preference at multiple positions surrounding the substrate cleavage site, rather than the recognition of a specific amino acid sequence, plays an important role for ADAM10-mediated cleavages (84).

We then exploited this new feature to assess levels of PrP^C^ shedding in different models. We found that it significantly varies among the animal models, with wild type mice showing the highest level of shed PrP^C^ and significantly more than bank voles and some transgenic mouse lines. Interestingly, among the transgenic mouse lines, we observed a significantly higher shedding in tg340 compared with tg361 mice, that is, two transgenic models sharing an identical shedding site, comparable levels of PrP^C^ expression, and the same human PrP^C^ sequence, except for residue 129, which represents a disease-relevant polymorphism. Albeit restricted by the shortcomings of a preliminary study conducted on a limited number of animals, such differences highlight a possible correlation between the level of constitutive PrP^C^ shedding and susceptibility to prion diseases. Indeed, bank voles and mice have a different susceptibility to prions and rather opposite prion strain preferences (65, 67, 70), whereas the genotype at codon 129 of human PrP^C^ is known to be critical for the risk of acquired and sporadic prion diseases as well as in the phenotype modification of prion diseases (85, 86, 87, 88). Moreover, bank voles and transgenic mice expressing bank vole PrP (*i.e.*, two models sharing identical PrP^C^ primary sequence, similar levels of expression but different genetic background) showed comparable levels of shed PrP^C^, indicating that murine ADAM10 operates preserving not only the exogenous PrP^C^ cleavage site but also the exogenous physiological processing of PrP^C^ of the respective species. Overall, these results suggest that the PrP^C^ sequence, rather than the overexpression of exogenous PrP^C^ or the genetic background, might influence PrP^C^ shedding rates, which deserves further analysis, particularly with regard to prion diseases.

Finally, the method also allowed the detection of the less abundant PrP^C^ fragments derived from γ-cleavage, recently reported in different cell lines, tga20 mouse brain homogenates, and human brain samples (48). Lewis *et al.* identified a 6-7 kDa C-terminal, GPI-anchored, and presumably nonglycosylated fragment, that they called C3, and another C-terminal fragment, C3′. Because of the detection of a single N-terminal fragment of approximately 20 kDa in size in PNGase F-untreated tga20 brain homogenates, that they named as N3, they concluded that γ-cleavage might not always occur on FL-PrP^C^, but rather on a precursor fragment, that is, C3′, and preferentially on a nonglycosylated substrate. We observed comparable C-terminal fragments in all the rodent models tested. However, while our results confirm that C3 is nonglycosylated as reported by Lewis *et al.*, we detected C3′ only upon PNGase F treatment, which implies the presence of N-linked sugars in this fragment. We also detected a nonglycosylated fragment corresponding to the one identified as N3 by Lewis *et al.* but, based on its glycosylation status and apparent MW of ∼17 kDa, we propose that it could represent the N-terminal counterpart of the glycosylated fragment C3′ and refer to this proteoform as N3′. In support of this, we observed another N-terminal yet glycosylated proteoform with an apparent MW of ∼20 kDa, frequently masked by the more abundant C2 fragment, which could indeed represent the N-terminal counterpart of C3, and we thus referred to this as N3. The different glycosylation status of C3 and C3′, the identification of two N-terminal fragments with opposite glycosylation status, and the unawareness of the exact cleavage site of γ-cleavage led us to hypothesize the presence of two alternative “γ-cleavage-like” sites, one N-terminal (C3′ + N3′) and one C-terminal (C3 + N3) to the two N-glycosylation sites of PrP^C^. Overall, these results show that γ-cleavage (or γ-cleavage-like events) is also present in bank voles and tg mouse lines expressing heterologous PrPs and suggest a physiological significance of this C-terminal processing of PrP^C^. The low abundance of these proteoforms, however, did not allow for a more detailed and quantitative assessment of γ-cleavage, which deserves further studies.

In conclusion, we report a new WB-based method able to detect all currently known major PrP^C^ proteoforms and to quantify the most abundant ones. This method will be useful to study the effect of molecules targeting the endoproteolytic processing of PrP^C^ and to compare PrP^C^ processing in different species, tissues, and cell lines, ultimately leading to a better understanding of these processes and their physiological roles. Importantly, this method allows for the first time to quantitatively assess PrP^C^ shedding and will thus be useful for gaining insight into pathophysiological roles, biomarker suitability in easy-to-assess body fluids (*e.g.*, cerebrospinal fluid or blood), and therapeutic potential of shed PrP^C^. Finally, a method that allows identifying and quantifying C1, C2, and sFL, together with FL-PrP^C^, will be useful for a comprehensive appraisal of PrP^C^ levels and possible alterations in proteoform composition in PrP^C^-lowering therapies or in the screening of such drugs, as well as for studying both possibly meaningful disease-related alterations in the proteoform pool and an influence of the PrP^C^ proteoforms on the progression of neurodegenerative diseases.

## Experimental procedures

### Rodent models

All transgenic mouse lines (reported in Table S1) are on PrP null background and are homozygous for the transgene. Cohorts (n  =  4, 6–8 weeks of age and sex matched) of each model were used. Bank voles, mouse, and tg mouse lines tg338, tg501, tg110, tg340, and tg361 were obtained from the breeding colony of Istituto Superiore di Sanità. Preliminary analysis conducted on bank vole carrying either methionine or isoleucine at PrP residue 109 showed no differences among the two lines; so for data homogeneity, all reported results were obtained using BV109Met (reported throughout the text as BV). The experimental protocol was approved and supervised by the Service for Biotechnology and Animal Welfare of the Istituto Superiore di Sanità and authorized by the Italian Ministry of Health (decree number: 1119/2015-PR). All procedures were carried out in accordance with European Council directives 86/609 and 2010/63 as well as in compliance with the Italian Legislative Decree 26/2014. Tga20, tg407, and tgBVΔGPI snap-frozen brain tissues were provided by Dr Altmeppen, Dr Torres, and Dr Castilla, respectively.

### PrP^C^ analysis

Mice were sacrificed by cervical dislocation. Tissue was immediately extracted, frozen on dry ice, and kept at −80 °C until use. For each sample, a hemisected brain tissue was homogenized as 20% w/v concentration in cold Tris–HCl at pH 7.4 with cOmplete Protease Inhibitor Cocktail (Roche) and maintained on ice for the whole procedure. In order to minimize the presence of unspecific proteolytic fragments that might interfere with this analysis, (i) the homogenates were aliquoted to avoid multiple freezing/thawing cycles and stored at −20 °C; (ii) PMSF was added in three steps, that is, during the first denaturation step, just before the addition of PNGase F, as it stops the majority of proteases active at 38 °C but not PNGase F, and during the second denaturing step before the loading of the sample in the gel. About 20 μl of brain homogenates were added with an equal volume of 100 mM Tris–HCl (pH 7.4) and sarcosyl 4% and incubated for 30 min at 37 °C with gentle shaking. As each transgenic mouse line was characterized by its own level of PrP^C^ expression, 20% w/v brain homogenates from transgenic mice were first diluted into 20% w/v brain homogenate from KO mice so to have PrP^C^ chemiluminescence signals roughly comparable to that of bank vole and then subjected to the same procedure. Samples were then added with NuPage LDS Sample Buffer (Invitrogen), completed with 50 mM dithiothreitol (NuPage Sample Reducing Agent; Invitrogen) and 6 mM PMSF (Sigma–Aldrich) to final concentration, and incubated for 10 min at 95 °C with gentle shaking. For the untreated samples, 40 μl of denatured samples were taken and stored at −20 °C, whereas 10 μl were subjected to deglycosylation treatment. Briefly, they were first added with 32 μl of 0.2 mM sodium phosphate buffer (pH 7.4) and gently shaked for 2 min at 38 °C, then added with 4 μl of Nonidet P-40 (Roche), and gently shaked for 2 min at 38 °C and finally added with 3 mM PMSF and 4 μl (80 U/ml) of *N*-glycosidase F (PNGase F; Roche) and incubated at 38 °C overnight with gentle shaking. Untreated and PNGase F-treated aliquots were diluted in complete NuPage LDS Sample Buffer in order to have a final brain homogenate concentration of 0.02 and 0.006 mg eq/μl respectively, heated for 10 min at 90 °C, and then 10 μl of each sample loaded on Nupage 12% Bis–Tris polyacrylamide gels (Invitrogen). Because of the low PrP^C^ expression level, for the analysis of tgBVΔGPI, 20 μl per lane were loaded. For the methanol concentration protocol, denatured samples were added with methanol (1:5), maintained at −20 °C for 1 h, and centrifuged at 15,000*g* for 15 min at 4 °C; the pellets obtained were resuspended in the needed volume of complete NuPage LDS Sample Buffer, heated for 10 min at 90 °C, and loaded on gel. To prevent sample reoxidation and maintain the proteins in a reduced state, electrophoresis was conducted in the presence of NuPage Antioxidant (Invitrogen), which was added in the running buffer of the Upper (cathode) Buffer Chamber. Samples were then analyzed by WB on polyvinylidene fluoride membranes (Trans-Blot Turbo Mini/Midi 0.2 μm Polyvinylidene Fluoride Transfer Pack; Bio-Rad) using the Trans-Blot Turbo Transfer System (Bio-Rad) selecting the “Mixed MW proteins” protocol. The membranes were processed with anti-PrP antibodies by using the SNAP i.d. 2.0 system (Millipore) according to the manufacturers’ instructions. After incubation with horseradish peroxidase–conjugated antimouse immunoglobulin (Pierce Biotechnology) at 1:20,000 dilution or anti-rabbit immunoglobulin (Thermo Fisher Scientific) at 1:10,000 dilution, PrP^C^ isoforms were visualized by enhanced chemiluminescent substrate (Pierce Biotechnology) and the ChemiDoc imaging system (Bio-Rad).

### Anti–prion protein antibodies

PrP-specific antibodies used for the epitope mapping assay were mouse mAbs 8B4 (Santa Cruz Biotechnology), SAF32 (AlfaTech), Sha31 (AlfaTech), and 12B2 (WBVR), rabbit mAb EP1802Y (Abcam), and rabbit polyclonal sPrP^G227^ (63). The epitopes (mouse sequence) of these antibodies are as follows: _37_RYPGQGSP_45_ for 8B4; PrP octarepeat for SAF32; _88_WGQGG_92_ for 12B2; _145_YEDRYYRE_152_ for Sha31 (human sequence); and _221_SQA_223_ and Y_225_ for EP1802Y (72). For the production of rb pAb sPrP^G227^, the immunogenic peptide _221_QAYYDG_227_-COOH (G-COOH represents G_227_ as the PrP C terminus exposed after ADAM10-mediated cleavage) has been used as described previously (63). Based on their respective epitopes, we can group these antibodies into four categories: (i) 8B4 and SAF32 that recognize the flexible N-terminal portion of PrP^C^; (ii) 12B2 that recognizes the internal part of PrP^C^ and whose epitope is between α- and β-cleavage sites; (iii) Sha31 and EP1802Y, which recognize the C-terminal part of PrP^C^; and (iv) sPrP^G227^ that exclusively detects shed PrP^C^ whose new C terminus is G_227_-COOH.

### PrP^C^ proteoform quantification

All the quantifications have been performed on samples treated with PNGase F using four individual brains for each rodent model, as shown in the representative WB in Fig. S5, and are expressed as mean ± SD. The chemiluminescence signal of each detected PrP^C^ proteoform was quantified by the Image Lab 6.1 software (Bio-Rad), using the “Lanes and Bands” tool in order to precisely isolate the bands.

For assessing the relative amount of the six most abundant proteoforms, it was necessary to combine data obtained with different mAbs. With this analysis, the relative abundance of FL-PrP^C^, sFL, C1, C2, N1, and N2 was assessed. Individual PrP^C^ proteoforms were previously quantified in all individual brains, relative to FL-PrP^C^ set as “1,” using mAb EP1802Y for quantifying sFL and C2, Sha31 for C1, and SAF32 for N1 and N2. Then, total PrP^C^ signal was obtained by summing the relative values of all proteoforms (including FL-PrP^C^ set as “1”). To obtain the percentage amount of the six assessed PrP^C^ proteoforms on the total PrP^C^ amount, we then divided the value obtained for each proteoform by total PrP and multiplied for 100.

The sFL/FL ratio was determined by calculating the chemiluminescence signal of the two bands detected with the same antibody. For this analysis, four individual animals for each rodent model were loaded in the same blot, and the analysis was repeated three times, thus obtaining four mean individual values for each rodent model that allowed to calculate the mean sFL/FL value ± SD. Statistical comparison of sFL/FL values of the rodent models analyzed was made with the statistical software Prism (GraphPad Software, Inc). One-way ANOVA (*p* < 0.05) and Tukey's multiple comparisons test were used to calculate significances.

## Data availability

All data generated or analyzed during this study are included in this published article and its supporting information files.

## Supporting information

This article contains supporting information (70, 89, 90, 91, 92, 93, 94, 95).

## Conflict of interest

The authors declare that they have no conflicts of interest with the contents of this article.
