# Supplementary material for: An optimized Western blot assay provides a comprehensive assessment of the physiological endoproteolytic processing of the prion protein
Source: J Biol Chem. 2022 Dec 22;299(2):102823. doi: 10.1016/j.jbc.2022.102823 (PMC9867980; doi:10.1016/j.jbc.2022.102823)
Supplement: Supporting information [file mmc1.pdf]

An optimized western blot assay provides a comprehensive assessment of the physiological endoproteolytic processing of the prion protein

Ilaria Vanni[1*], Floriana Iacobone[1], Claudia D'Agostino[1], Matteo Giovannelli[1], Laura Pirisinu[1], Hermann Clemens Altmeppen[2], Joaquin Castilla[3,4], Juan Maria Torres[5], Umberto Agrimi[1], Romolo Nonno[1]

[1]Department of Food Safety, Nutrition and Veterinary Public Health, Istituto Superiore di Sanità, Rome, Italy
[2]Institute of Neuropathology, University Medical Center Hamburg-Eppendorf, Martinistraße 52, 20251 Hamburg, Germany
[3]Basque Research and Technology Alliance (BRTA) - CIC BioGUNE & IKERBasque, Bizkaia, Spain
[4]Centro de Investigación Biomédica en Red de Enfermedades infecciosas (CIBERINFEC), Instituto de Salud Carlos III, Madrid, Spain
[5]Centro de Investigación en Sanidad Animal (CISA-INIA-CSIC), 28130, Valdeolmos, Madrid, Spain
[*]Corresponding author

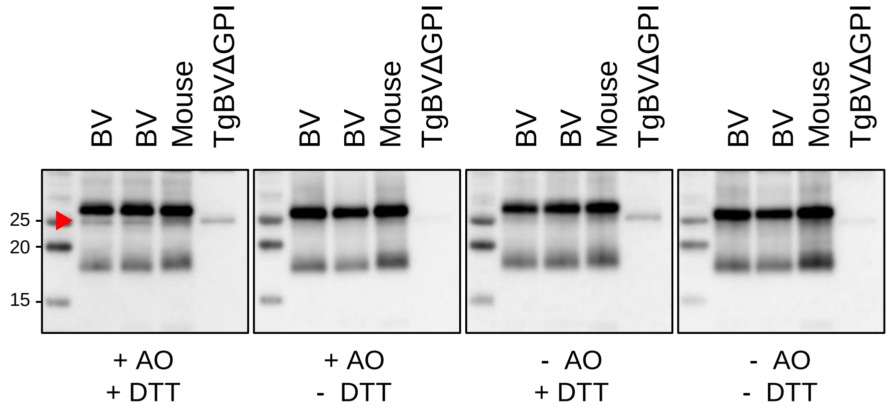

Fig. S1. Reduction of PrP^C disulphide bonds is necessary for FL-PrP^C and shed FL-PrP^C discrimination. Representative western blots of PNGase F treated brain homogenates prepared from 2 bank voles, one wild type mouse and the transgenic mouse line expressing BV PrP devoid of the GPI anchor (tgBVΔGPI). Samples were loaded in the presence (+DTT, added into the sample buffer before the denaturation step) or absence (-DTT) of the reducing agent, with the addition (+AO) or not (-AO) of antioxidant agent in the running buffer of the Upper Buffer Chamber. The red solid arrowhead indicates shed FL-PrP^C (sFL). sFL was detected only in presence of the reducing agent (first and third blot), but it was clearly distinguishable from FL-PrP^C only in the presence of both the reducing and antioxidant agents (+AO/+DTT, first blot of the panel). Molecular weight markers (25, 20 and 15 kDa) were loaded in the first lane of each blot and the respective kilodaltons reported on the left. Tissue equivalent (TE) loaded per lane was 0.06 mg. Membranes were probed with mAb 9A2.

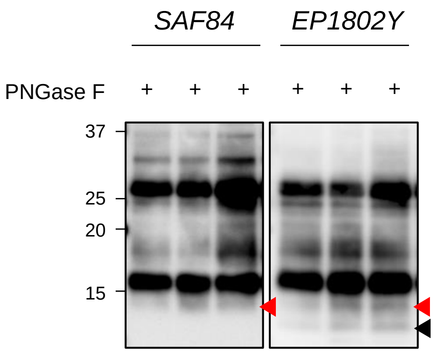

Fig. S2. Characterization of N3’↓C3’ gamma-cleavage-like site. Representative western blots of PNGase F treated brain homogenates prepared from three bank voles. Replica blots were probed with the antibodies SAF84 and EP1802Y (indicated at the top of each membrane). The SAF84 epitope ($_{160}$VYYRPVDQY$_{169}$ bank vole sequence) is fully maintained in sC1 (red arrowhead), while it is absent in C3’ (black solid arrowhead), indicating that the cleavage site producing the fragments N3’ and C3’ is located between SAF84 and EP1802Y epitopes. The positions of the molecular weight markers and the respective kilodaltons (37, 25, 20 and 15 kDa) are reported on the left. Tissue equivalent (TE) loaded per lane was 0.06 mg.

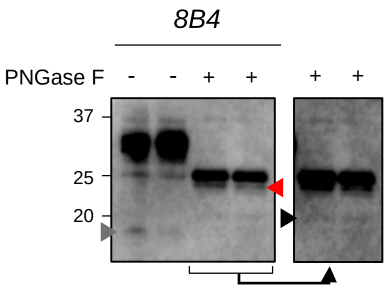

Fig. S3. Identification of the N-terminal PrP^{C} fragments generated by gamma-cleavage-like processing. Representative western blot of brain homogenates prepared from two bank voles subjected to PNGase F treatment ('+', third and fourth lanes of the blot) or left untreated ('-', first two lanes of the blot) and analysed with the extreme N-terminal antibody 8B4. N3' (grey arrowhead, apparent MW of 17 kDa) was detected only in untreated samples, while N3 (black arrowhead, apparent MW ~20 kDa) was detected only upon PNGase F treatment, suggesting a different glycosylation status for the two fragments. A long exposure (black arrow, the bracket indicates the portion of the blot shown after a longer exposure) was necessary for a clearer identification of N3. Molecular weight markers (37, 25 and 20 kDa) and the respective kilodaltons are reported on the left. Tissue equivalents (TE) loaded per lane were 0.2 mg ('-' samples) and 0.06 mg ('+' samples). The red solid arrowhead in PNGase F treated samples indicates sFL.

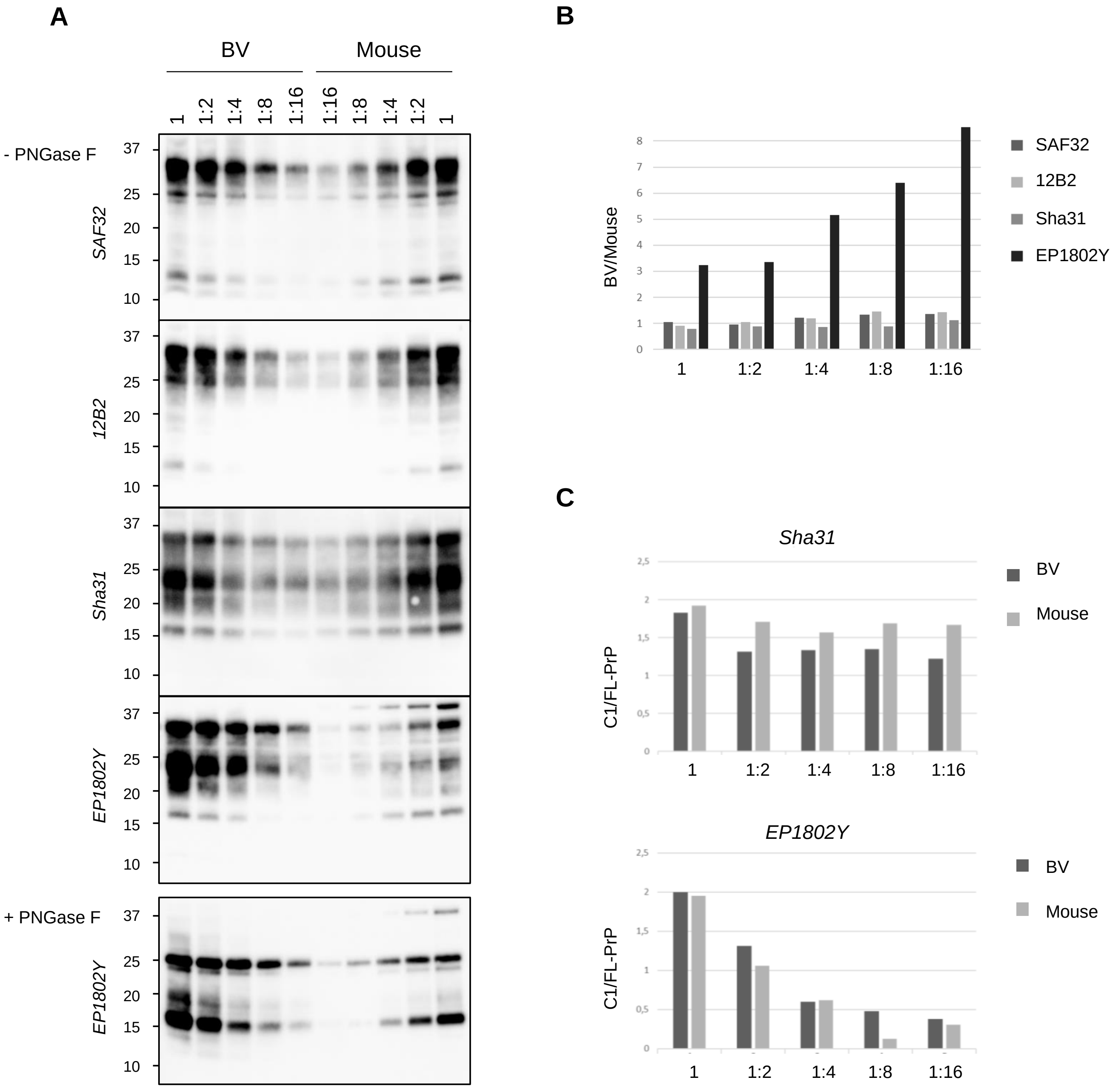

Fig. S4. Analysis of mAbs reactivity in wild type rodent models. A) Representative western blots of a 2-fold dilution curve of brain homogenates prepared from wild type mouse and bank vole left untreated or PNGase F treated as indicated on the right. '1' stands for the undiluted sample (0.2 mg of tissue equivalent for untreated samples, 0.06 mg for PNGase F treated samples). Replica blots were probed with different mAbs, indicated on the left of each blot. EP1802Y was used on both untreated and PNGase F-treated 2-fold

dilution curves. Bank vole and mouse PrPs were equally detected by Abs SAF32, 12B2 and Sha31 but not by EP1802Y, which better recognises bank vole PrP^C. A comparable difference is detected also in PNGase F treated dilution curves. Molecular weight markers (37, 25, 20, 15, 10 kDa) and the respective kilodaltons are reported on the left. **B)** Graph depicting the BV/mouse chemiluminescence signal ratio at each point of the dilution curves of both wild type mouse and bank vole untreated samples, detected by Abs SAF32, 12B2, Sha31 and EP1802Y. Mouse and bank vole PrPs are similarly detected by SAF32, 12B2 and Sha31 as indicated by a BV/mouse ratio of approximately 1, independently of the dilution analysed. However, the ratio turns up to 3 with EP1802Y, and it increases with increasing dilutions of the models, indicating that the affinity of EP1802Y for bank vole PrP^C is higher than for mouse PrP^C. **C)** Graphs depicting the C1/FL-PrP^C chemiluminescence signal ratio at each point of the dilution curve of both wild type mouse and bank vole PNGase F treated samples, detected by Abs EP1802Y or Sha31. While comparable C1/FL-PrP^C values were obtained at each point of the dilution curve with Sha31, with EP1802Y a decrease of the values was observed with increasing dilutions in both models, indicating that the affinity of EP1802Y for C1 is lower than for FL-PrP^C.

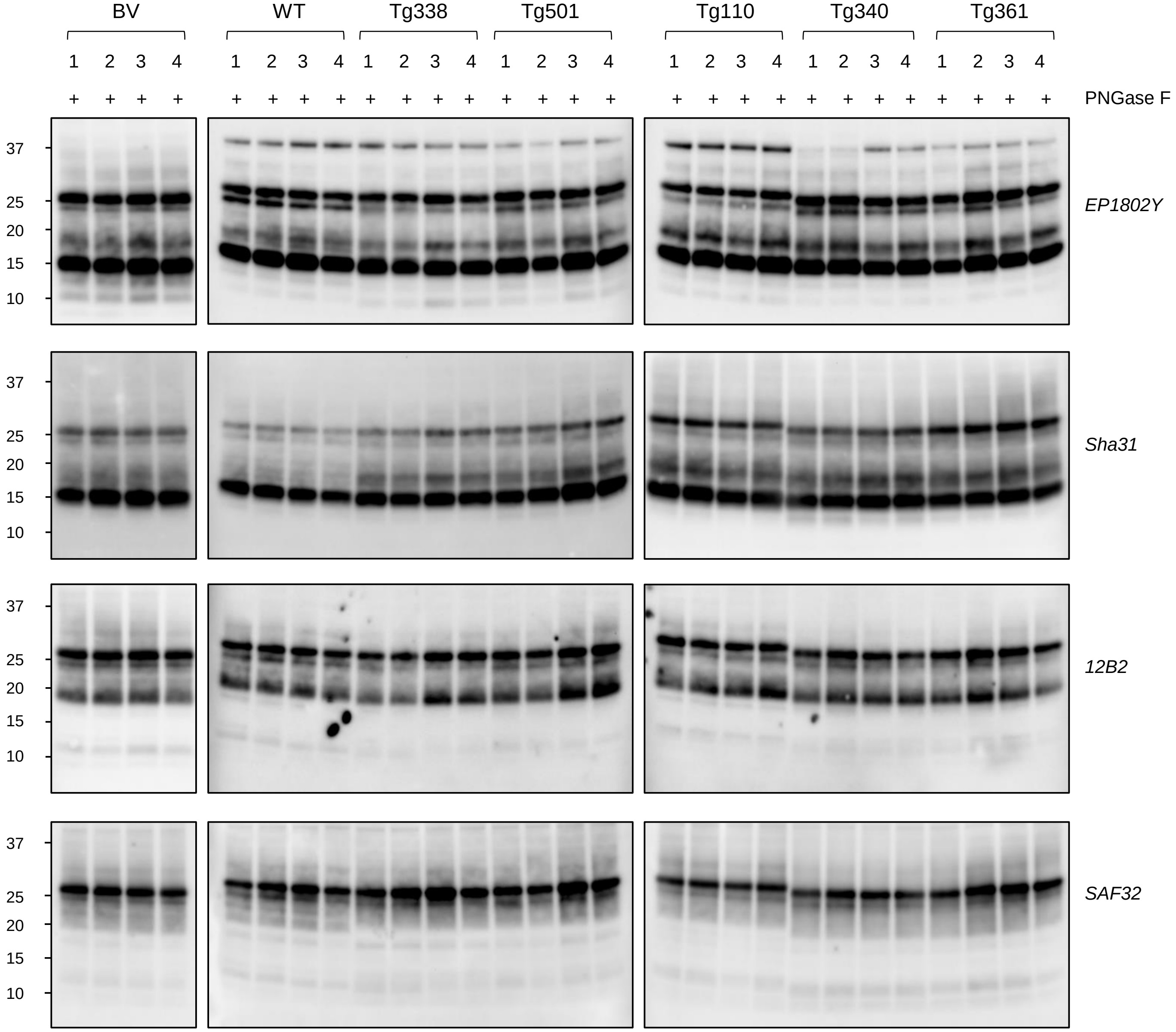

Figure S5. Detailed analysis of PrP^C proteoforms present in the brain of wild type and transgenic rodent models. Representative western blots of PNGase F treated brain homogenates prepared from bank vole (BV), wild type (WT), tg338, tg501, tg110, tg340 and tg361 mice. To evaluate inter-individual variability in terms of PrP^C proteoform pattern, 4 individuals per model were analysed. Replica blots were probed with different mAbs, indicated on the right of each panel. Tissue equivalent (TE) loaded per lane was 0.06 mg. MW markers (37, 25, 20, 15 and 10 kDa) and the respective kilodaltons are reported on the left.

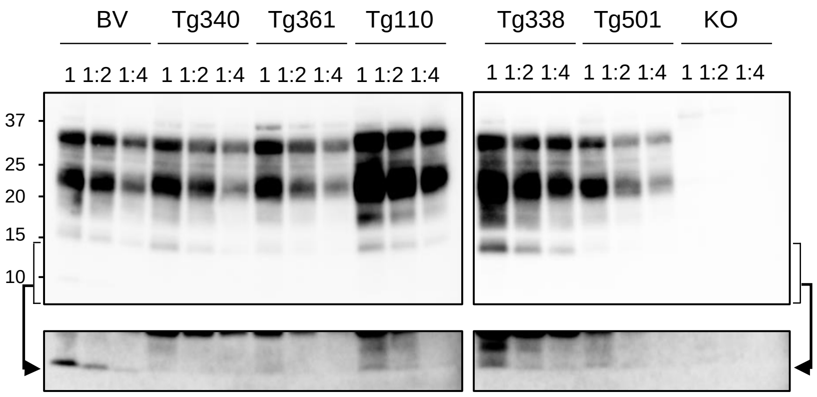

Figure S6. Identification of C3 in transgenic mouse lines. Representative western blots of 2-fold dilution curves of untreated brain homogenates prepared from bank vole, tg340, tg361, tg110, tg338, tg501 and PrP-KO mice. ‘1’ stands for the undiluted sample (0.2 mg of tissue equivalent loaded). A long exposure (black arrow, the bracket indicates the portion of the blot shown after a longer exposure) was necessary to evaluate the presence of C3. C3 was clearly detected in bank vole brain even at the last point of the dilution curve (lanes 1 to 3), but a comparable detection of C3 was missing in all transgenic mice, independently from the level of heterologous PrP^{C} expression. MW markers (37, 25, 20, 15 and 10 kDa) and the respective kilodaltons are reported on the left.

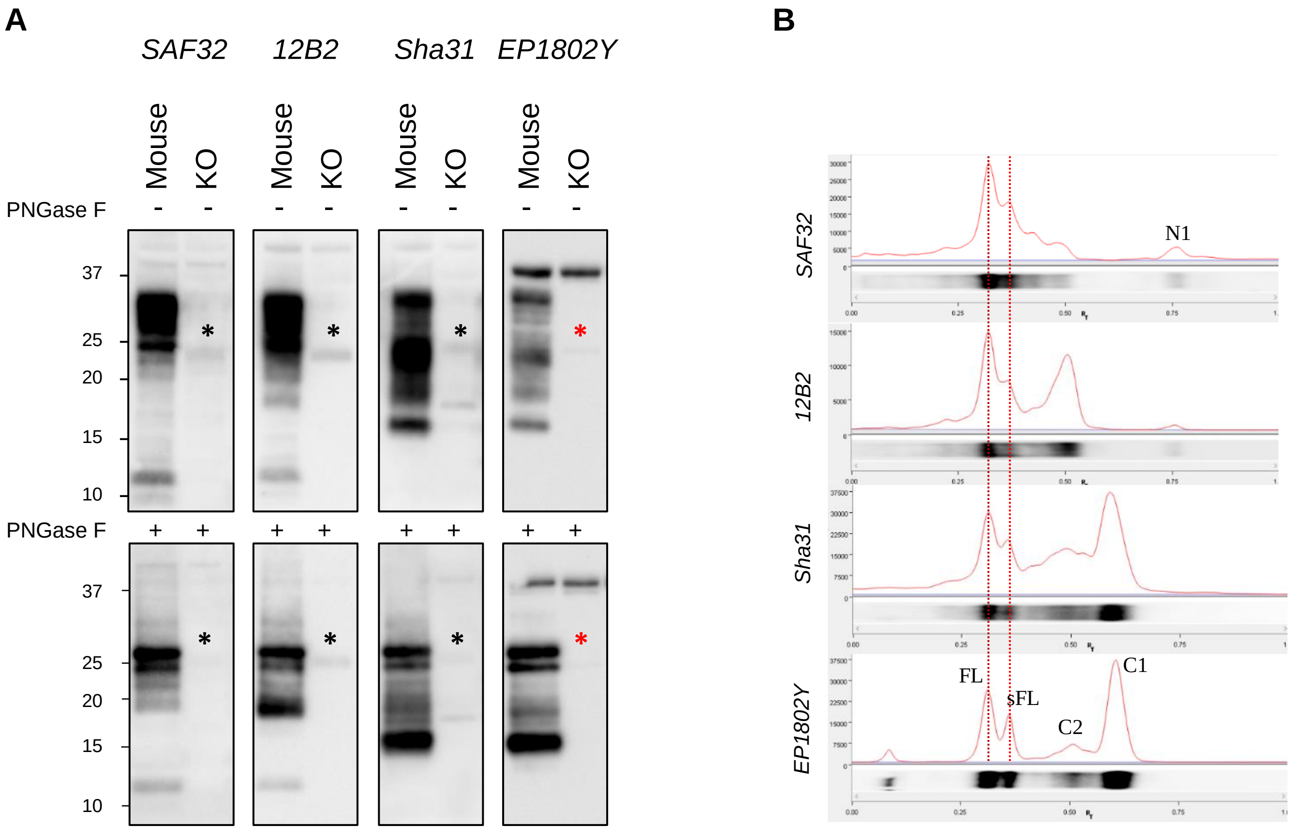

Figure S7. The presence of non-specific bands might affect FL-PrP^C/shed PrP^C discrimination and quantification. A) Representative western blots of brain homogenates prepared from wild type mouse and tg PrP-KO left untreated ('- PNGase F', upper blots of the panel) or PNGase F treated ('+ PNGase F', lower blots of the panel). Replica blots were probed with different Abs, indicated at the top of each pair. Tissue equivalents (TE) loaded per lane were 0.2 mg ('-' samples) and 0.06 mg ('+' samples). The black asterisks indicate the non-specific fragment detected by the anti-mouse secondary antibody used for SAF32, 12B2 and Sha31 analysis. Despite being very faint after deglycosylation treatment due to the lower TE loaded per lane (compare lanes 2, 4 and 6 of the upper blots with their counterparts of the lower blots), the non-specific fragment is still detectable and localized between the FL-PrP^C and shed FL bands detected in the wild type. A non-specific fragment is also detected by the anti-rabbit secondary antibody used for EP1802Y (red asterisk), but it became almost undetectable upon deglycosylation. MW markers (37, 25, 20, 15 and 10 kDa) and the respective kilodaltons are reported on the left. **B)** Lane profile of PNGase F-treated wild type mouse brain homogenate subjected to western blot and detected with

Abs SAF32, 12B2, Sha31 and EP1802Y (lanes 1, 3, 5 and 7 of the lower blots in figure A). The most abundant PrP^C fragments in each lane are indicated. FL-PrP^C (FL) and shed FL (sFL) are detected by the whole set of Abs, as indicated by the two close peaks on the left in each lane profile. Note that FL and sFL chemiluminescence signal partially overlap when analysed with SAF32, 12B2 and Sha31 due to the presence of the non-specific fragment detected by the anti-mouse secondary antibody, while two clearly distinct peaks are obtained with EP1802Y.

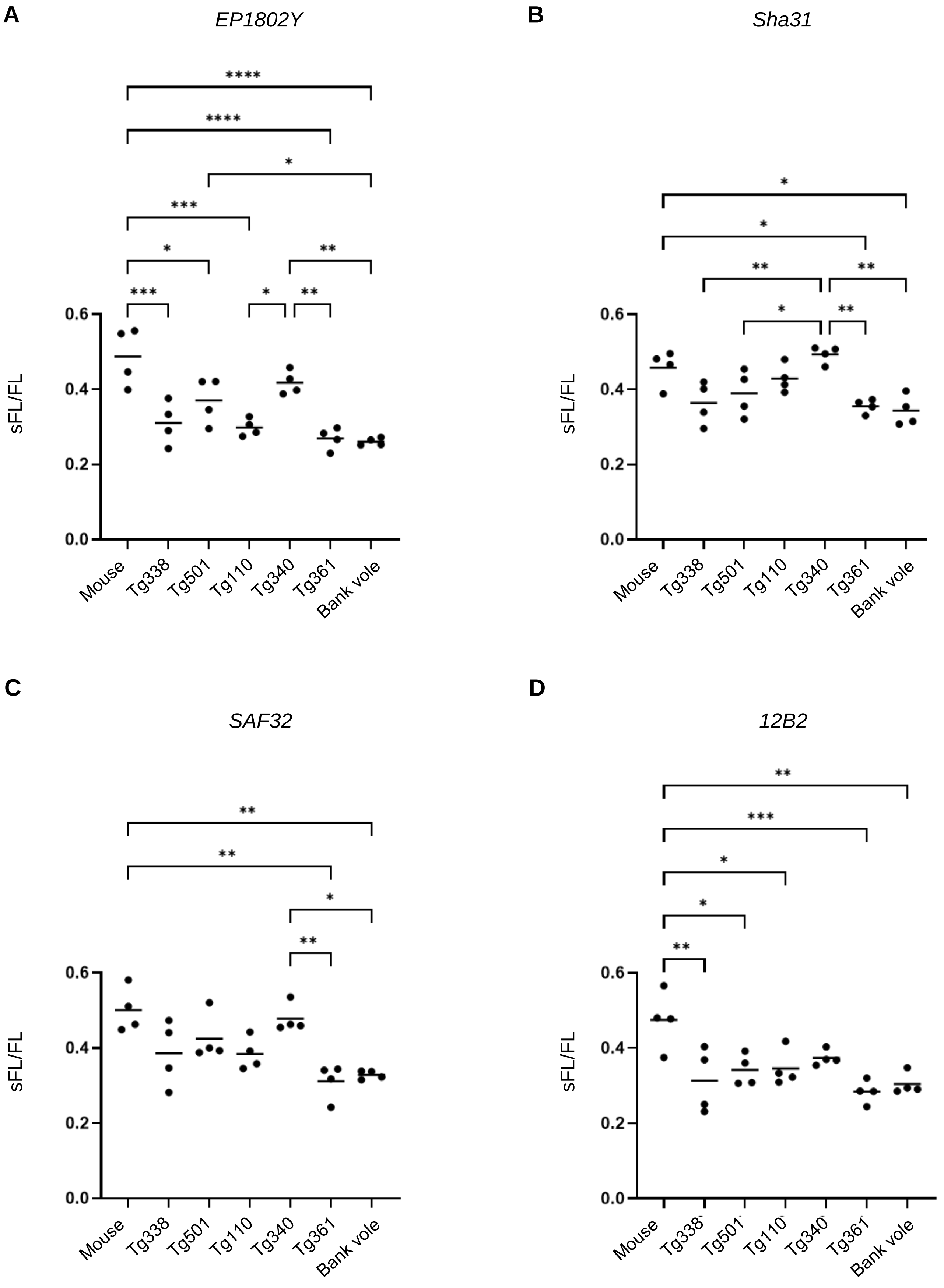
A
EP1802Y
B
Sha31
C
SAF32
D
12B2
sFL/FL
0.0
0.2
0.4
0.6
Mouse
Tg338
Tg501
Tg110
Tg340
Tg361
Bank vole
*
**
***
****

Fig. S8. Quantitative assessment of shed PrP^{C} in wild type and transgenic rodent models. Graph depicting the sFL/FL ratio in the set of rodent models analyzed. The ratio was determined by calculating the chemiluminescence signal of FL-PrP^{C} and shed FL (sFL) detected by mAb EP1802Y (**A**), Sha31 (**B**), SAF32 (**C**) and 12B2 (**D**) in 4 individuals per model. The analysis was repeated 3 times. Each black dot in the graph represents the value of a single individual resulting from the mean of 3 independent experiments, while the bar represents the mean for each model. One-way ANOVA showed significant variability among the models in all the datasets (Sha31: $F = 6.817$, $p = 0.0004$, $R2 = 0.661$; 12B2: $F = 5.646$, $p = 0.0013$, $R2 = 0.617$; SAF32: $F = 6.741$, $p = 0.0004$, $R2 = 0.658$). Tukey's multiple comparisons test generally confirmed the data obtained with EP1802Y, although showing less marked differences in pairwise comparisons, probably due to the interference of the non-specific fragment detected by the anti-mouse secondary antibody (used for the mouse monoclonal anti-PrP mAbs Sha31, 12B2 and SAF32). *, **, *** and **** indicate $p < 0.05$, $p < 0.01$, $p < 0.001$ and $p < 0.0001$, respectively.

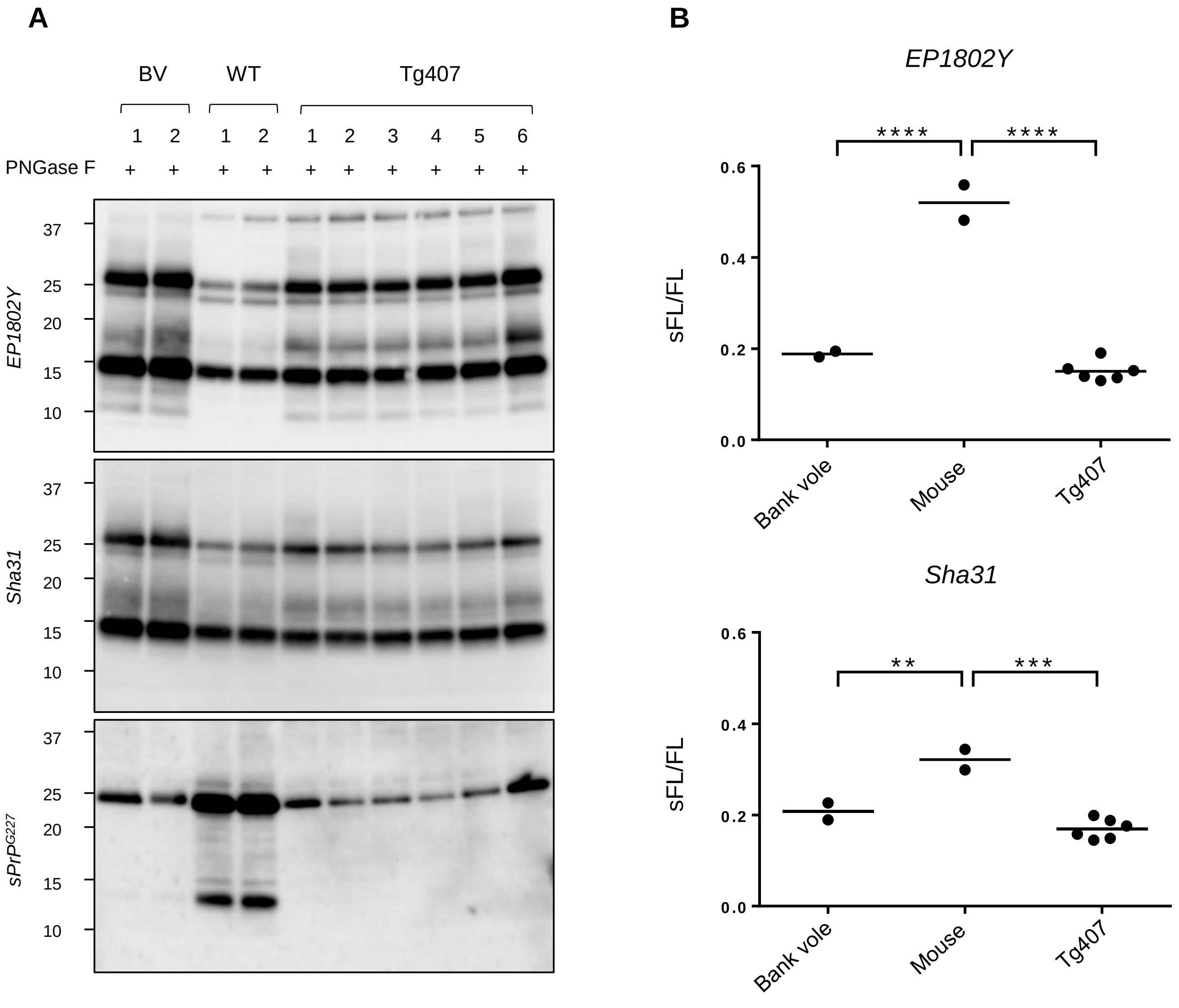

Fig. S9. Comparison of shed FL-PrP^C in bank voles, wild type and tg mice expressing bank vole PrP. A) Representative western blots of PNGase F treated brain homogenates prepared from bank voles (BV), wild type (WT) and tg407 (expressing bank vole PrP) mice. For tg407, six individuals were analysed. Replica blots were probed with different Abs, indicated on the left of each panel. Tissue equivalent (TE) loaded per lane was 0.06 mg. MW markers (37, 25, 20, 15 and 10 kDa) and the respective kilodaltons are reported on the left. Note that only tg407 #6 is $BVPrP^{+/+}$, while all the other individuals are $BVPrP^{+/-}$. **B)** Graph depicting the sFL/FL ratio in the set of rodent models analyzed. The ratio was determined by calculating the chemiluminescence signal of $FL\text{-}PrP^{C}$ (FL) and its shed counterpart (sFL) detected by mAbs EP1802Y and Sha31 in the individuals (2 bank voles, 2 mice, 6 tg407) analysed in the blot in **A**. Each black dot in the graph represents the value of a single individual, while the bar represents the mean for each model. One-way

ANOVA showed significant variability among the models in the two datasets (EP1802Y: $F = 135.2$, $p < 0.0001$, $R^2 = 0.9748$; Sha31: $F = 29.69$, $p = 0.0004$, $R^2 = 0.8945$). **, *** and **** indicate $p = 0.0055$, $p = 0.0003$, $p < 0.0001$, respectively, in pairwise comparison (Tukey's multiple test).

Table S1. Main features of the rodent models used in this study

Line	Species	PrP sequence	Reported PrP expression level	References
**Tga20**	Mouse	Mouse - Prnp allele a	10X	(89)
**Tg338**	Mouse	Sheep-VRQ	8X	(90)
**Tg501**	Mouse	Goat-ARQ	2X	(91)
**Tg110**	Mouse	Bovine	8X	(92)
**Tg340**	Mouse	Human-129MM	4X	(93)
**Tg361**	Mouse	Human-129VV	4X	(94)
**TgBVΔGPI**	Mouse	Bank vole	0.2X	Unpublished, provided by Dr. Castilla
**Tg407**	Mouse	Bank vole	1X	(70)
**KO**	Mouse	absent	-	(95)
